# Effects of long-term metal exposure on the structure and co-occurrence patterns of the oral microbiota of residents around a mining area

**DOI:** 10.3389/fmicb.2023.1264619

**Published:** 2023-10-19

**Authors:** Shuwei Pei, Lu Feng, Yonghua Zhang, Jiangyun Liu, Jia Li, Qiwen Zheng, Xingrong Liu, Bin Luo, Ye Ruan, Huan Li, Weigang Hu, Jingping Niu, Tian Tian

**Affiliations:** ^1^School of Public Health, Lanzhou University, Lanzhou, Gansu, China; ^2^School of Stomatology, Lanzhou University, Lanzhou, Gansu, China; ^3^Child Health Department, Lanzhou Maternal and Child Health Care Hospital, Lanzhou, Gansu, China; ^4^State Key Laboratory of Herbage Improvement and Grassland Agro-Ecosystems, College of Ecology, Lanzhou University, Lanzhou, China

**Keywords:** heavy metal, buccal mucosa, bacteria, keystone taxa, network analysis, 16S rRNA gene sequencing

## Abstract

**Objectives:**

The aim of our study was to investigate the impact of long-term exposure to heavy metals on the microbiome of the buccal mucosa, to unveil the link between environmental contamination and the oral microbial ecosystem, and to comprehend its potential health implications.

**Methods:**

Subjects were divided into two groups: the exposure group and the control group. We collected samples of buccal mucosa, soil, and blood, and conducted microbial diversity analysis on both groups of oral samples using 16S rRNA gene sequencing. The concentrations of heavy metals in blood and soil samples were also determined. Additionally, microbial networks were constructed for the purpose of topological analysis.

**Results:**

Due to long-term exposure to heavy metals, the relative abundance of *Rhodococcus*, *Delftia*, *Fusobacterium*, and *Peptostreptococcus* increased, while the abundance of *Streptococcus*, *Gemella*, *Prevotella*, *Granulicatella*, and *Porphyromonas* decreased. The concentrations of heavy metals in the blood (Pb, Cd, Hg, and Mo) were associated with the growth of *Rhodococcus*, *Delftia*, *Porphyromonas*, and *Gemella*. In addition, the relative abundances of some pathogenic bacteria, such as *Streptococcus anginosus*, *S. gordonii*, and *S. mutans*, were found to be enriched in the exposure group. Compared to the exposure group network, the control group network had a greater number of nodes, modules, interactive species, and keystone taxa. Module hubs and connectors in the control group converted into peripherals in the exposure group, indicating that keystone taxa changed. Metals in the blood (Pb, Cd, Hg, and Mo) were drivers of the microbial network of the buccal mucosa, which can have adverse effects on the network, thus providing conditions for the occurrence of certain diseases.

**Conclusion:**

Long-term exposure to multiple metals perturbs normal bacterial communities in the buccal mucosa of residents in contaminated areas. This exposure reduces the complexity and stability of the microbial network and increases the risk of developing various diseases.

## 1. Introduction

As one of the most important components of the national economy over the past several decades, the mining industry has played a major role in the rapid industrial transformation of China. However, due to a number of factors (e.g., lack of pollution control and ineffective enforcement of regulations), the metal mining industry has caused severe heavy metal contamination (Chen L. et al., 2022; Shi et al., 2022). These metal pollutants not only cause serious damage to natural resources such as land, water, and air but also pose a great threat to human health (Shao and Zhu, 2020).

Metal pollution is covert, persistent, and irreversible (Ghnaya et al., 2015). Once uncontrolledly discharged into soil and water, metals can persist for a long time (Li et al., 2014; Xia et al., 2019). Heavy metals that have been discharged accumulate in soil and water and eventually enter the human body through the food chain or in direct contact with the skin (Liu et al., 2013; Azimi et al., 2017). High concentrations of lead (Pb) can damage the human blood and nervous system (Ahamed and Siddiqui, 2007; Kumar et al., 2020). Mercury (Hg) can damage the nervous system when present in excess amounts (Driscoll et al., 2013), while cadmium (Cd) can impair kidney function and even cause cancer (Matović et al., 2015; García-Pérez et al., 2016). In addition, recent research has shown that heavy metal pollution also affects the structure of human microbial communities (Shao and Zhu, 2020; Zhang et al., 2023).

The oral cavity is one of the earliest organs in the body to be exposed to the external environment, and its internal microbiome is more susceptible to changes due to external factors such as diet, medicines, pollutants in the environment, and geographical location (Thomas et al., 2014; Dong et al., 2021; Gupta et al., 2022). Oral microbial communities are closely related to human health, affecting not only oral health but also the health of the whole body (Gao et al., 2018; Zhang Y. et al., 2018). Currently, it is believed that a number of diseases, including dental caries and periodontal diseases, are associated with imbalances in oral microorganisms (Wade, 2013; Curtis et al., 2020; Hajishengallis and Lamont, 2021). According to a previous report (Zhang et al., 2023), heavy metal pollution altered the abundance and diversity of the oral microbiome of mining residents and promoted the development of periodontitis. Studies have also shown that oral microbial disorders can lead to diabetes, Alzheimer’s disease, cardiovascular disease, and cancer (Sudhakara et al., 2018; Irfan et al., 2020; Zhang et al., 2020). In addition, because they are at the beginning of the digestive tract, oral microbes have a close connection with intestinal microbes. Some oral bacteria were able to enter the gut through the enteral route or hematogenous route, affecting the intestinal microbiome and immune responses (Lu et al., 2023). For instance, *Porphyromonas gingivalis*, an oral anaerobic bacterium in the oral cavity, altered the composition of the intestinal microbiota when administered orally in mouse experiments (with an increased proportion of Bacteroidetes and a decreased proportion of Firmicutes), and this alteration is considered to be attributed to the increase of the serum endotoxin level resulting from *P. gingivalis* infection (Nakajima et al., 2015). Simultaneously, *P. gingivalis* also induces inflammation in intestinal tissues and disrupts the ratio of the T-helper 17 cell/T-regulatory cells in the colon (Wang T. et al., 2022).

Exposure to heavy metals can affect the structure of microbial communities in the oral cavity. Although many studies have investigated this topic in recent years, most have been limited to saliva and plaque samples. However, the oral mucosa is an important component of the oral cavity, has an extremely rich microbial population, and is closely associated with a variety of diseases (Chen J. et al., 2022; Wang S. et al., 2022). Therefore, studying changes in the structure of microbial communities in the oral mucosa under the influence of metal exposure is important for us to gain a deeper understanding of the effects of heavy metal exposure on oral microbial communities and the occurrence of related diseases.

Baiyin City, located in the province of Gansu, China, is known as the “Chinese Copper City” because of its rich resources of nonferrous metals. Due to the frequent mining activities of the last century, heavy metals have been released into the environment, causing severe pollution in the soil, water sources, and air (Yue et al., 2020). Contaminated crops, vegetables and animal foods enter the human body through the food chain, eventually causing heavy metals to accumulate in the local residents (Li et al., 2006; Zhao et al., 2020). In previous studies, the harmful effects of metal exposure on human and animal health in the area have been reported (Zhang et al., 2016; Zhang Q. et al., 2018). However, the effect of metal exposure on the human oral mucous microbiome is still unclear. Therefore, this paper reports the effects of long-term metal exposure on the microbial community structure and co-occurrence patterns of the oral mucous of local residents, filling the gap in epidemiological research on the interaction of heavy metals and buccal mucosal bacteria.

## 2. Materials and methods

### 2.1. Study area

Due to long-term nonferrous metal smelting and mining in the last century, the air, soil, surface water, and groundwater of Baiyin city, Gansu Province, are seriously polluted. We selected two adjacent villages in the region as representative contaminated areas: Minqin village and Shuanghe village (36°28′38.188″ N, 104°18′47.870″ E; 36°27′24.650″ N, 104°21′22.057″ E). For comparison, we selected another two adjacent villages, namely, Hewan village and Yangwa village (35°46′41.541″ N, 104°0′37.443″ E; 35°45′54.661″ N, 104°1′28.117″ E), located in Yuzhong County of Lanzhou City as control areas, which are 100 km away from Baiyin City and characterized by relatively low levels of heavy metal pollution. The two selected regions have similar levels of socioeconomic development and residents with similar lifestyles and dietary habits.

### 2.2. Collection of soil samples and heavy metal analysis

**Figure 1:**
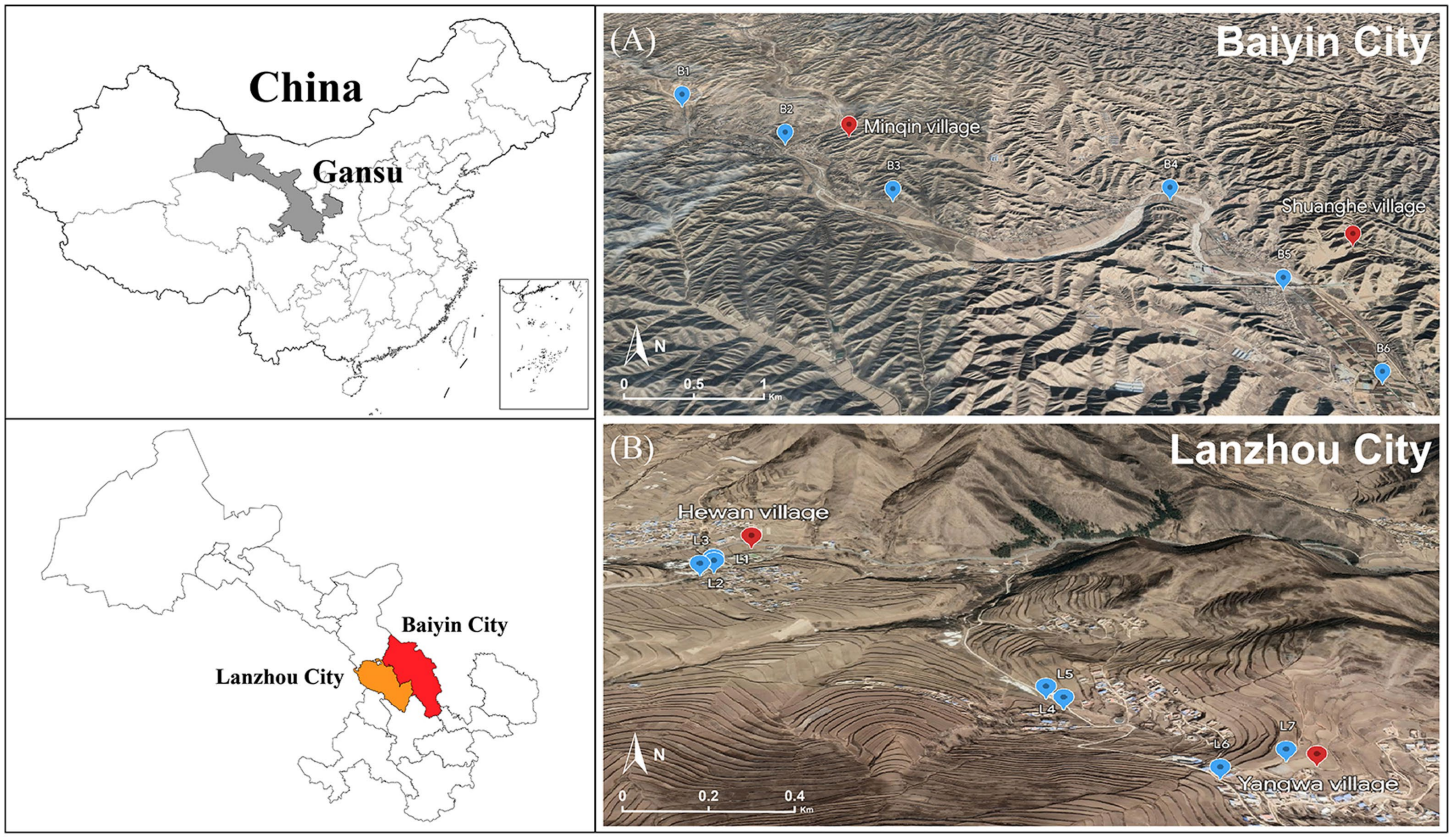
Location of sampling points in the contaminated **(A)** and control areas **(B)**.

Soil samples were collected in April 2021 from the contaminated and control areas to assess the levels of heavy metal pollution. A total of 13 sampling points were selected in this study (B1–B6, L1–L7), with B1–B6 located in the field in the vicinity of Minqin village and Shuanghe village (Figure 1A) and L1–L7 located in the field near Yangwa village and Hewan village (Figure 1B). At each sampling point, areas of approximately 10 × 10 meters were randomly selected in the fields, and five subsampling sites were set up in each selected field using a five-point sampling method. After removing gravel and impurities at the surface, soil from five subsampling points (at 20 cm depths) was collected using a sterile wooden spatula and thoroughly mixed into a composite sample. A total of 13 samples were collected. The samples were sent to the laboratory on the same day. To determine the heavy metal content in soil, each soil sample was first air-dried at room temperature, and then biological debris, plant roots, leaves, and stones were removed, followed by sieving through a 200-mesh nylon sieve. Finally, each sample was thoroughly mixed and stored in a polyethylene bag for further analysis. Each sample of approximately 0.5 g was digested using a microwave digestion system (Sartorius, PB-10, Germany). Then, the content of heavy metals (Mn, Sb, Cu, Cd, Zn, Hg, Pb, Mo, Co, and Ni) was measured using inductively coupled plasma–mass spectrometry (ICP–MS, Agilent, United States). Quality assurance/control procedures were conducted using standard reference materials (Chinese Academy of Measurement Science) with each batch of samples (one blank and one standard).

### 2.3. The collection of oral buccal mucosa and blood samples

In this study, a total of 137 subjects were enrolled for the collection of both buccal mucosa and blood samples. Among these, 92 subjects were from Baiyin City (i.e., Minqin and Shuanghe villages), and the remaining 45 were from Yuzhong County (i.e., Hewan and Yangwa villages). Subjects were further divided into two groups according to heavy metal exposure: (1) the exposure group (*n* = 92), comprised of residents residing in Baiyin District; and (2) the control group (*n* = 45), comprised of residents residing in Yuzhong District. The enrolled subjects had an average age of 60.03 ± 6.47 years (mean ± SD; range 42–72 years old). All participants fulfilled the following four criteria: (i) subjects signed informed consent and had not used any antibiotics for at least 3 months prior to sampling; (ii) subjects had no oral diseases, such as halitosis, chronic xerostomia, untreated cavitated caries lesions, abscesses, cancer, or candidiasis; (iii) subjects who reported being ill or unwell on the day of sampling were excluded; and (iv) subjects had at least 24 teeth. Prior to sample collection, the participants were restrained from drinking or eating and were asked to wash their mouth with drinking water 30 min before samples were taken. Then, using a sterile cotton-wool swab, we scraped the buccal mucosa on the left and right sides of their mouths for 10 s. After oral sampling, all samples were preserved immediately at −80°C until subsequent processing in the laboratory.

To investigate the association between the concentration of heavy metals in the blood and the composition of the buccal mucosa microbiota, blood samples were collected from the peripheral veins of some of the enrolled research subjects (*n* = 79). We collected 15 mL of heparinized venous blood, removed 2 mL of whole blood, and stored it at −80°C. The contents of heavy metals in blood were measured using an inductively coupled-mass spectrometer (ICP–MS, Elan DRC-II ICP–MS, PerkinElmer Sciex, United States). Survey subjects agreed to informed consent, and the study was approved by the Ethical Committees of the Public Health School of Lanzhou University.

### 2.4. DNA extraction, sequencing and bioinformatic analyses

DNA was extracted from each buccal mucosa sample using an E.Z.N.A. Soil DNA Kit (Omega Bio-Tek, Norcross, GA, United States) following the manufacturer’s instructions, and its concentration and purity were assessed on a 1% agarose gel. The hypervariable region V3–V4 of the bacterial 16S rRNA gene were amplified with primer pairs 338F (5′-ACTCCTACGGGAGGCAGCAG-3′) and 806R (5′-GGACTACHVGGGTWTCTAAT-3′) by an ABI GeneAmp® 9700 PCR thermocycler (ABI, CA, United States). Thermocycling conditions consisted of 3 min at 95°C followed by 30 amplification cycles of 30 s denaturation at 95°C, 30 s annealing at 55°C, 72°C for 45 s, and a final extension of 72°C for 10 min. All amplification reactions were performed in a total volume of 20 μL containing 4 μL of 5× FastPfu Buffer, 2 μL of 2.5 mM dNTPs, 0.8 μL of both the forward and reverse primers, 10 ng of template DNA, and 0.4 μL of FastPfu DNA Polymerase. To mitigate individual PCR biases, each sample was amplified in triplicate and pooled together. The amplicon quality of the PCR products was assessed on a 2% agarose gel, followed by purification with an AxyPrep Gel Extraction Kit (Axygen Biosciences, United States). Purified amplicons were combined at equimolar concentrations and paired-end sequenced (2 × 300 bp) on an Illumina MiSeq platform (Illumina, United States) at the Majorbio Bio-pharm Technology Co., Ltd. (Shanghai, China) according to standard protocols. Raw sequencing data of the bacterial 16S rRNA gene have been deposited in the NCBI Sequence Read Archive under BioProject accession number PRJNA979792. The resulting sequences were processed using the QIIME pipeline (Caporaso et al., 2010). Briefly, low-quality sequences were trimmed with Cutadapt and quality-filtered. Paired-end reads were assembled using FLASH version 1.2.11 (Magoč and Salzberg, 2011). USEARCH was used to remove chimeric sequences based on the UCHIME algorithm (Edgar et al., 2011), and the remaining sequences were allocated to operational taxonomic units (OTUs) with 97% similarity using the UPARSE pipeline. OTUs with fewer than two sequences were eliminated, and their representative sequences were assigned to taxonomic lineages using the RDP classifier version 2.2 (Wang et al., 2007) against the SILVA database (version 138) using confidence threshold of 0.7.

### 2.5. Construction and analysis of the bacterial molecular ecological network of the buccal mucosa

The bacterial molecular ecological networks (MENs) of the buccal mucosa were constructed using an online tool called the Molecular Ecosystem Network Analysis Pipeline (MENAP; Feng et al., 2022).^1^ In the process of network construction, OTUs with a frequency of less than 10% were discarded. Based on the SparCC method with the default parameters, the filtered OTU table was utilized to calculate pairwise correlation (Friedman and Alm, 2012). Based on random matrix theory (RMT), the appropriate cut-off value was selected as a threshold and combined with a significance level of *p-*value adjusted using the Benjamini-Hochberg FDR correction method less than 0.05 to filter out unrelated associations in the matrix. The IDIRECT method was used to remove unreliable and indirect associations from the network (Xiao et al., 2022). After networks were built, the analysis of network properties [e.g., nodes, links, average degree (avgK), average path distance (GD), average clustering coefficient (avgCC), connectedness (Con), and modularity], and randomization were carried out using the default parameters (Deng et al., 2012). Network modules were then determined by using greedy modularity optimization. In this study, a total of three networks were constructed. Among them, networks with sample sizes of 92 and 45 were employed for the comparison between the exposure (*n* = 92) and control (*n* = 45) groups. Additionally, to investigate the impact of blood heavy metals on network structure, a network with a sample size of 79 was utilized to calculate the correlation between module-based eigenvalues and the concentration of heavy metals in blood. Furthermore, the functions of the modules were predicted by PICRUSt (Douglas et al., 2020) and the Kyoto Encyclopaedia of Genes and Genomes (KEGG) database (Kanehisa et al., 2017) in terms of metabolic pathways. All obtained networks were visualized using Gephi 0.9.7^2^ and Cytoscape 3.9.1.^3^

### 2.6. Statistical analyses

Prior to analyses, the OTU table was subsequently rarefied to the lowest number of sequences (28367) found within an individual sample. Our resampled dataset included a total of 2,315 bacterial OTUs. We first calculated the α diversity index (i.e., Sobs, Shannon–Wiener, Simpson, ACE and Chao1 indices) for each buccal mucosa sample using the QIIME pipeline and then tested the differences between the exposure and control groups using the Wilcoxon rank-sum test. Principal coordinate analysis (PCoA) was then performed at OTU level to investigate the dissimilarities in bacterial community composition between the groups based on both weighted and unweighted UniFrac algorithms, and statistical significance was assessed by analysis of similarities (ANOSIM). We also compared the relative abundance of dominant bacterial taxa at both the phylum and genus levels (phyla with relative abundance above 1% and genera with relative abundance above 3% were considered dominant) between the groups using the Wilcoxon rank-sum test, and their *p* values were adjusted by using the false discovery rate. To identify the taxa most likely to account for the variations between exposure and control samples, we employed linear discriminant analysis effect size (LEfSe) analysis. An LDA score of greater than 3.5 was established as the threshold to identify discriminative taxa (from phylum to species). The Spearman correlation between the genera and the concentrations of heavy metals in blood was analyzed and visualized to further investigate whether the genera exhibiting differences between the exposed and control groups were correlated with the concentrations of heavy metals in blood. To assess the functional differences in terms of metabolic pathways between the microbial communities of the exposure and control groups, we used PICRUSt2^4^ based on the SILVA database of 16S rRNA sequences (Langille et al., 2013) and the Kyoto Encyclopaedia of Genes and Genomes (KEGG) database (Kanehisa et al., 2017) to predict microbial functional genes. The Wilcoxon rank-sum test was used for comparison of the KEGG pathway abundances between the two groups. All above analyses were conducted by using SPSS (version 26.0; IBM SPSS Inc., United States) and R (version 4.2.2; http://cran.r-project.org/).

## 3. Results and discussion

### 3.1. The heavy metal pollution of the study area

To assess whether there are differences in metal pollution levels between the contaminated and control areas, the concentrations of heavy metals in the soil and the blood of the subjects in the two areas were compared using the Wilcoxon rank-sum test. In the ploughed soil of the contaminated area, our results showed that the mean values of seven metals (Mo, Cd, Sb, Cu, Hg, Pb, and Zn) were substantially higher than those of the control area (all *p* < 0.05), whereas the levels of Co, Ni, and Mn were similar between the two areas (all *p* > 0.05; Supplementary Table S1). The concentrations of four metals (Zn, Hg, Cd and Pb) in the blood of subjects living in contaminated areas were significantly higher than those in the control area (all *p* < 0.05; Supplementary Table S2).

### 3.2. Bacterial diversity of buccal mucosa

**Figure 2:**
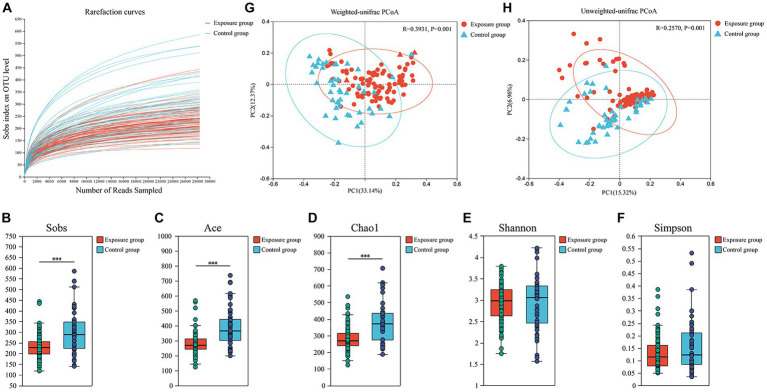
Analysis of α-diversity and principal coordinate analysis of buccal mucosal bacterial communities. **(A)** Rarefaction curves for the bacterial community dataset. The flat curve indicated that the sequence number used for analyses was adequate. **(B–F)** Differences in bacterial α diversity indices between the exposure and control groups. Significant differences were determined by using the Wilcoxon rank-sum test. ^*^p ≤ 0.05; ^**^p ≤ 0.01; ^***^p ≤ 0.001. **(G,H)** Principal coordinate analysis (PCoA) of bacterial community dissimilarities based on the weighted **(G)** and unweighted **(H)** UniFrac distances. Significant differences in bacterial β diversity between the exposure and control groups were determined by using ANOSIM statistics.

The rarefaction curves stabilized, indicating that the sequencing depth was sufficient to reflect the bacterial diversity in the majority of samples (Figure 2A). The bacterial Sobs, Chao1 and ACE indices of the exposure group were significantly higher than those of the control group (all *p* < 0.001), whereas we did not observe significant differences between the two groups for the Shannon–Wiener and Simpson indices (both *p* > 0.05; Figures 2B–F). The bacterial community structure of the buccal mucosa samples was analyzed using principal coordinate analysis (PCoA), which is based on both weighted and unweighted UniFrac distances, to see whether there were significant differences between the exposure and control groups. The first and second principal components together explained 45.51% and 22.28% of the total variation in bacterial communities based on weighted and unweighted UniFrac distances, respectively (Figures 2G,H). Our results revealed significant differences in bacterial community composition between the two groups (ANOSIM R > 0.257, both *p* = 0.001), although their distributions were found to partially overlap.

### 3.3. Bacterial community structure of buccal mucosa

**Figure 3:**
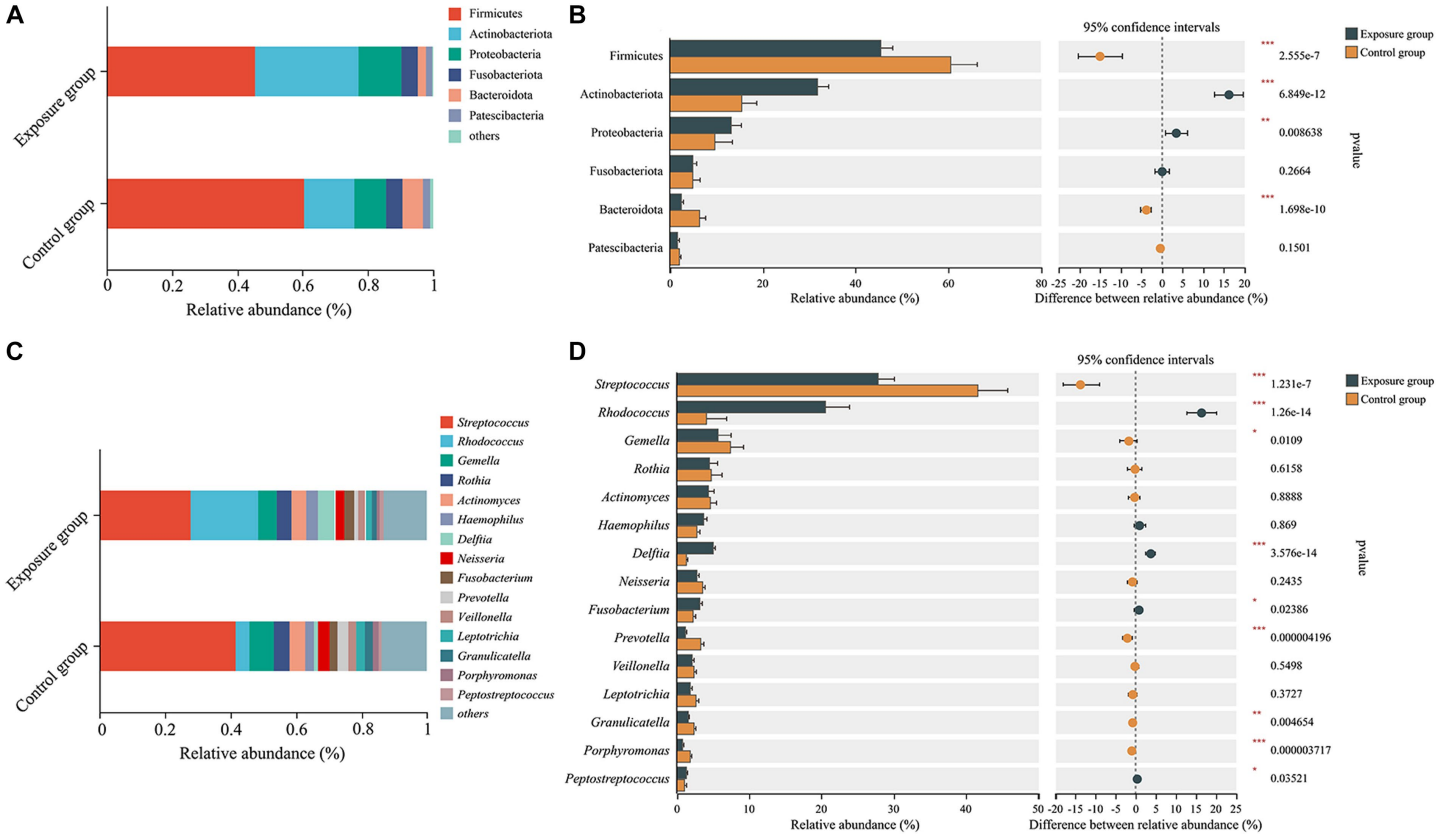
The bacterial community composition of the exposure and control groups and the correlations between bacterial community composition and blood heavy metal levels. Compositional differences in bacterial communities of the buccal mucosa at the phylum **(A,B)** and genus **(C,D)** levels. The *p*-value was calculated using the Wilcoxon rank-sum test and adjusted by using the false discovery rate. ^*^p < 0.05; ^**^p < 0.01; ^***^p < 0.001.

The dominant bacterial phyla observed in the exposure and control groups were Firmicutes (45.41% vs. 60.45%), Actinobacteriota (31.75% vs. 15.54%), Proteobacteria (13.19% vs. 9.74%), Fusobacteriota (5.00% vs. 4.98%), Bacteroidota (2.50% vs. 6.30%), and Patescibacteria (1.74% vs. 2.16%; Figure 3A). The relative abundances of Actinobacteriota and Proteobacteria were significantly higher in the exposure group than in the control group (Wilcoxon rank-sum test, both *p* < 0.01), whereas the relative abundances of Firmicutes and Bacteroidota appeared to be significantly higher in the control group (both *p* < 0.001; Figure 3B). Moreover, the dominant bacterial genera in the control group were *Streptococcus* (41.64%), *Gemella* (7.45%), *Rothia* (4.72%), *Actinomyces* (4.71%), *Rhodococcus* (4.18%), *Neisseria* (3.53%), and *Prevotella* (3.34%), while the exposure group exhibited dominant genera including *Streptococcus* (27.86%), *Rhodococcus* (20.57%), *Gemella* (5.71%), *Delftia* (5.09%), *Rothia* (4.53%), *Actinomyces* (4.40%), *Haemophilus* (3.70%), and *Fusobacterium* (3.13%; Figure 3C). Among the aforementioned dominant genera, the relative abundances of *Streptococcus*, *Gemella*, and *Prevotella* were significantly higher in the control group than in the exposure group (all *p* < 0.05), whereas those of *Rhodococcus*, *Delftia*, and *Fusobacterium* were significantly higher in the exposure group (all *p* < 0.05; Figure 3D). No significant differences were found in the relative abundance of *Rothia*, *Actinomyces*, *Haemophilus*, and *Neisseria* between the two groups. Furthermore, bacteria of the genera *Granulicatella* and *Porphyromonas* were significantly enriched in the control group (both *p* < 0.001), while *Peptostreptococcus* exhibited significant enrichment in the exposure group (*p* < 0.05).

**Figure 4:**
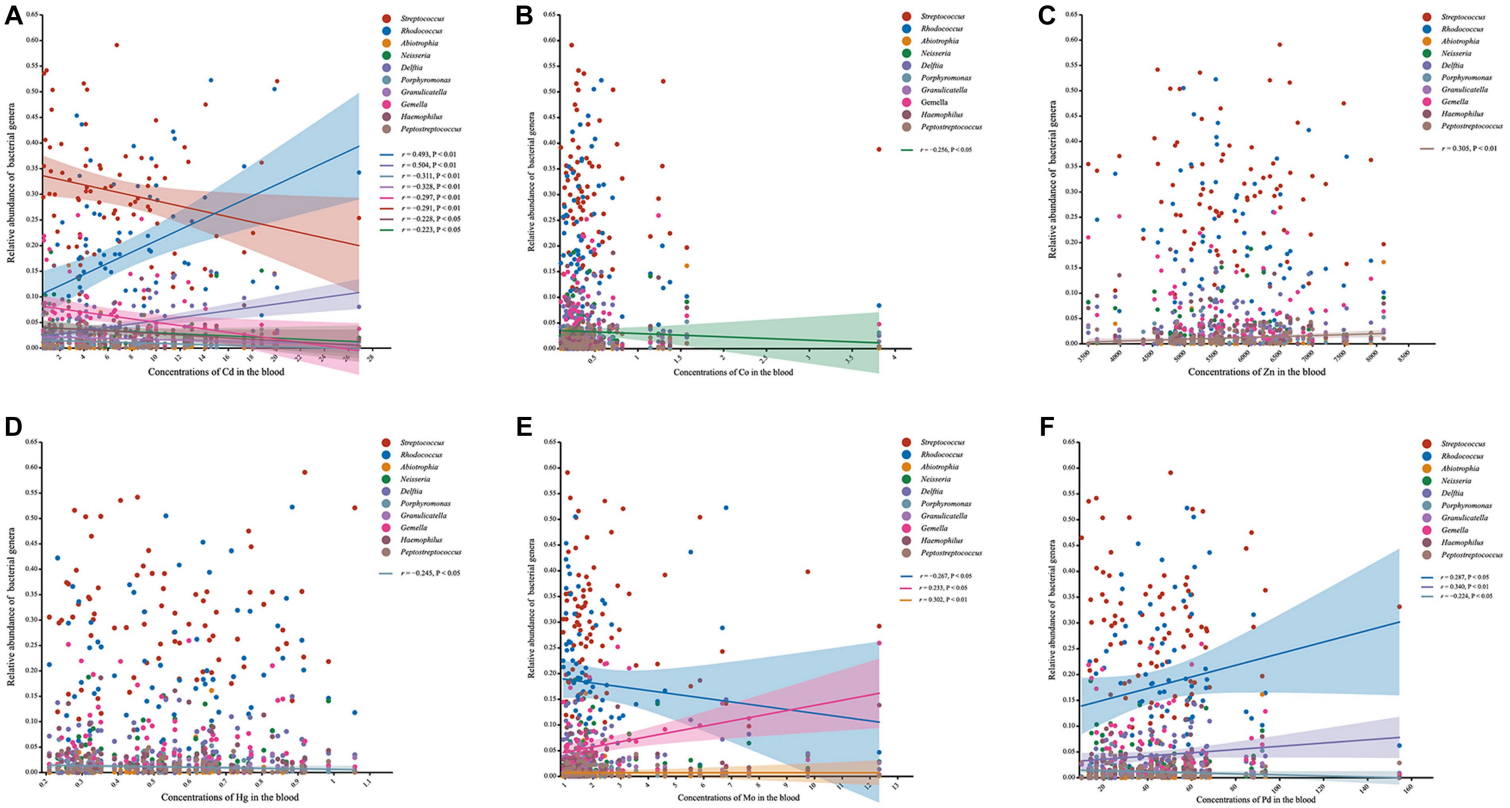
Scatter plots presenting the correlation between the genera and the concentrations of heavy metals in blood. **(A)** Scatter plot of the correlation between the genera and Cd. **(B)** Scatter plot of the correlation between the genera and Co. **(C)** Scatter plot of the correlation between the genera and Zn. **(D)** Scatter plot of the correlation between the genera and Hg. **(E)** Scatter plot of the correlation between the genera and Mo. **(F)** Scatter plot of the correlation between the genera and Pd.

We further used Spearman’s correlation to evaluate the responses of the relative abundance of bacterial genera to concentrations of heavy metals in the blood (Figure 4). Our results showed that the relative abundances of *Rhodococcus* and *Delftia* were positively correlated with the concentrations of Cd and Pb, whereas those of *Granulicatella*, *Streptococcus*, *Neisseria*, *Gemella*, *Haemophilus*, and *Porphyromonas* exhibited negative associations with the concentration of Cd. *Porphyromonas* exhibited not only a negative correlation with Cd but also negative correlations with Hg and Pb. Moreover, the relative abundance of *Peptostreptococcus* was found to be positively correlated with the amount of Zn, and those of *Gemella* and *Abiotrophia* showed positive correlations with the content of Mo. There were no significant associations observed between the relative abundances of *Prevotella*, *Leptotrichia*, *Fusobacterium*, *Rothia*, *Actinomyces*, *norank_f_Saccharimonadaceae*, and *Veillonella* with the concentrations of any heavy metals in the blood.

A portion of the bacteria at the species level are presented in Supplementary Figure S1. We found that the relative abundances of *Rhodococcus erythropolis*, *Delftia tsuruhatensis*, and *Streptococcus anginosus* in the exposure group were significantly higher than those in the control group. The relative abundances of *Streptococcus gordonii*, *S. gordonii*, *S. mutans*, and *Porphyromonas gingivalis* were enriched in the exposed group, although the difference was not significant compared to the control group.

Linear discriminant analysis effect size (LEfSe) analysis was used to identify taxa effect size. The circles that radiate outwards from the center of the branch diagram represent the various levels of classification, from phylum to species (Supplementary Figure S2). These results showed significant enrichment of *Corynebacteriales*, *Rhodococcus*, *Nocardiaceae*, Actinobacteriota, and *Actinobacteria* in the exposure group. We also found that *Streptococcus*, *Bacilli*, Firmicutes, *Lactobacillales*, and *Streptococcaceae*, were more abundant in the control group than in the exposure group (Supplementary Figure S3). Furthermore, Firmicutes, which is thought to be the most prevalent phylum of bacteria in the buccal mucosa (Wang S. et al., 2022), was significantly decreased in the exposure group. However, the cladogram for this phylum suggests that *Streptococcus*, *Gemella*, and *Granulicatella* were primarily accountable for this difference, and other genera belonging to the Firmicutes phylum did not show a preference for metal exposure.

### 3.4. Functional predictions of buccal mucosal bacterial communities

**Figure 5:**
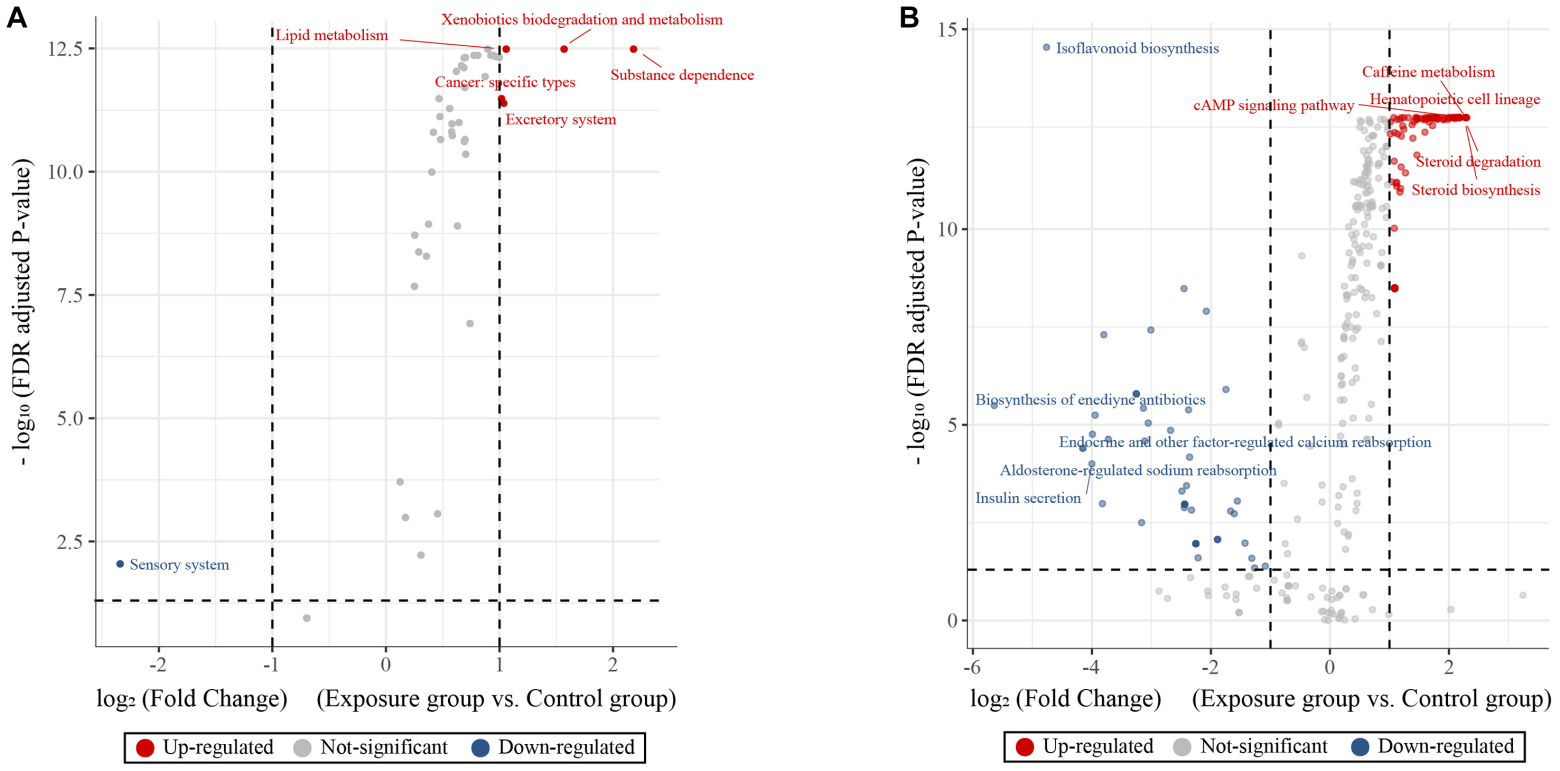
KEGG pathway volcano to predict differences in metabolic pathways of microbial communities. Volcano plot showing the predictive functional differences between the exposure and control groups based on the KEGG metabolic pathway at the second **(A)** and third **(B)** levels (fold change > 1 and adjusted *p*-value < 0.05). The red, blue and gray circles indicate upregulated, downregulated and insignificantly changed genes, respectively.

We predicted the functions of buccal mucosal bacterial communities based on the KEGG pathway database and then tested their functional differences in terms of metabolic pathways between the exposure and control groups (Figure 5). Overall, the two groups showed obvious functional differences, with a greater number of upregulated genes than downregulated genes (43 vs. 1 at KEGG level 2 and 258 vs. 64 at KEGG level 3). Specifically, predictions based on the KEGG level 2 pathway revealed that the top five upregulated genes in terms of fold change were involved in substance dependence, xenobiotic biodegradation and metabolism, lipid metabolism and catabolism, excretory system, and cancer: specific types were upregulated in the exposure group (Wilcoxon rank-sum test, all *p* < 0.05; Figure 5A), while sensory system-related genes were upregulated in the control group (*p* < 0.05). For the KEGG level 3 pathway, the top five upregulated genes in terms of fold change observed in the exposure group were involved in hematopoietic cell lineage, steroid degradation, caffeine metabolism, steroid biosynthesis and cAMP signaling pathway (Wilcoxon rank-sum test, all *p* < 0.05; Figure 5B). In contrast, the upregulated genes in the control group were involved in biosynthesis of enediyne antibiotics, isoflavonoid biosynthesis, aldosterone-regulated sodium reabsorption, endocrine and other factor-regulated calcium reabsorption, and insulin secretion (all *p* < 0.05).

### 3.5. Patterns of bacterial co-occurrence networks

**Figure 6:**
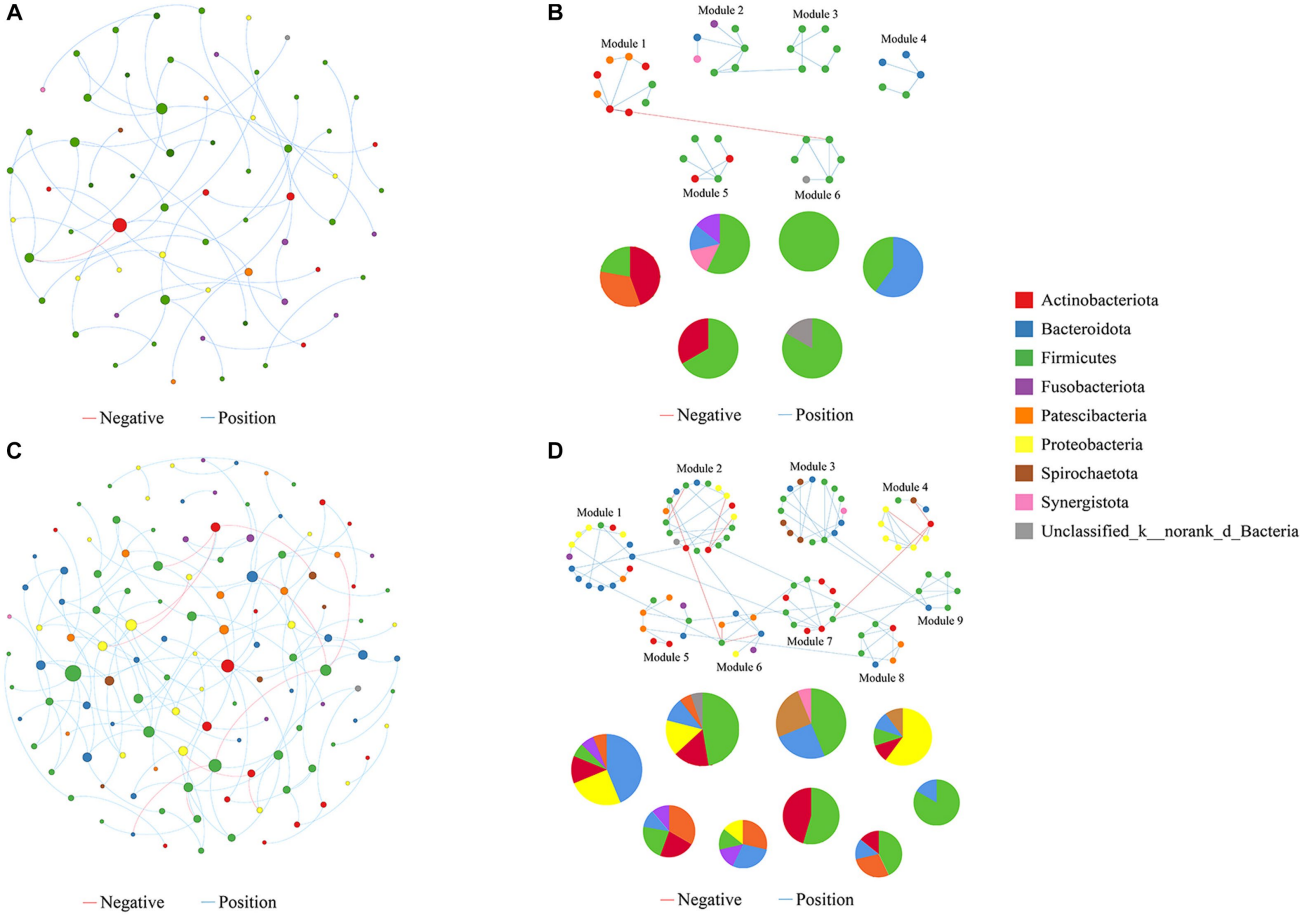
Molecular ecological networks were built on the basis of correlation among bacterial OTUs. The networks for the exposure **(A)** and control groups **(B)** are displayed. Node sizes are proportional to the number of connections. Panels **(C,D)** present the network modules determined by the fast greedy modularity optimization method (only showing nodes larger than 4) for the exposure and control groups, respectively, as well as their taxonomic composition at the phylum level. Each node represents a bacterial OTU and is colored by its phylum-level taxonomic affiliation. Red lines represent negative interactions among bacterial OTUs, whereas blue lines represent positive interactions.

We constructed molecular ecological networks (MENs) to compare the interaction and co-occurrence pattern of buccal mucosal bacterial communities between the exposure and control groups (Figure 6; Table 1). The network of the exposure group produced 66 nodes and 56 edges, while the network of the control group included 123 nodes and 135 edges (Table 1). For the network edges of the two groups, the proportion of positive interactions was much higher than that of negative interactions (98.21% vs. 1.79% in the exposure group, and 91.85% vs. 9.15% in the control group). To ascertain whether the networks of the two groups differ from random networks, we compared the empirical network with the random network generated using the Maslov-Sneppen procedure by rewiring the same number of nodes and edges to the corresponding empirical network (Zhou et al., 2011). The values of average clustering coefficient (avgCC), average path distance (GD), and modularity in both networks appeared to be significantly different from random ones (Supplementary Table S3), indicating nonrandom patterns of co-occurrence network.

**Table 1:** Topological properties of the empirical and 100 random MENs of microbial communities in the exposure and control groups; n.a denotes no data available in the random algorithm.

Network indices	Exposure group	Control group	Exposure group	Control group
	Empirical		Random (mean ± SD)	
Total nodes	66	123	n.a	n.a
Total links	56	135	n.a	n.a
RMT cut-off	0.45	0.45	n.a	n.a
R square of power-law	0.992	0.941	n.a	n.a
Average degree (avgK)	1.697	2.195	n.a	n.a
Average clustering coefficient (avgCC)	0.075	0.072	0 ± 0.005	0 ± 0.002
Average path distance (GD)	3.021	6.838	4.611 ± 0.928	5.814 ± 0.331
Geodesic efficiency (E)	0.460	0.199	0.321 ± 0.05	0.218 ± 0.009
Harmonic geodesic distance (HD)	2.174	5.027	3.188 ± 0.49	4.589 ± 0.199
Centralization of degree (CD)	0.082	0.048	0.082 ± 0	0.048 ± 0
Centralization of betweenness (CB)	0.058	0.246	0.16 ± 0.063	0.194 ± 0.048
Centralization of stress centrality (CS)	0.058	0.523	0.004 ± 0.002	0.004 ± 0.002
Centralization of eigenvector centrality (CE)	0.920	0.934	0.921 ± 0.015	0.89 ± 0.027
Centralization of closeness centrality (CCL)	0.006	0.015	0.014 ± 0.004	0.019 ± 0.004
Density (D)	0.026	0.018	0.026 ± 0	0.018 ± 0
Reciprocity	1	1	1 ± 0	1 ± 0
Transitivity (Trans)	0.146	0.065	0.014 ± 0.021	0.014 ± 0.012
Connectedness (Con)	0.136	0.675	0.311 ± 0.088	0.753 ± 0.065
Efficiency	0.887	0.984	0.999 ± 0.001	0.998 ± 0
Hierarchy	0	0	0.026 ± 0	0.018 ± 0
Lubness	1	1	0.142 ± 0.045	0.085 ± 0.018
Modularity	0.848 (17)	0.793 (18)	0.799 ± 0.018	0.725 ± 0.014

Network topological properties revealed that degree distributions conformed to the power-law model (both *R*^2^ > 0.941; Table 1), indicating the scale-free property of the two networks. The empirical networks exhibited higher average clustering coefficients compared to their corresponding random networks, which suggested the small-world property of the two networks (Deng et al., 2012). Modularity was utilized as a quantitative measure to evaluate the degree to which a network is organized into delimited modules. The modularity values for the exposure group (0.848) and the control group (0.793) were higher than for the corresponding random network (0.799 and 0.728), indicating that the two networks are modular (Newman, 2006; Deng et al., 2012). There were 6 and 9 modules (with >4 nodes) observed in the exposure and control groups, respectively (Figures 6C,D). Not only is the number of modules in the exposure group reduced compared to the control group pattern, but there are also fewer nodes and links within each module. These results suggested that the two networks possessed scale-free, small-world, and modular properties.

**Figure 7:**
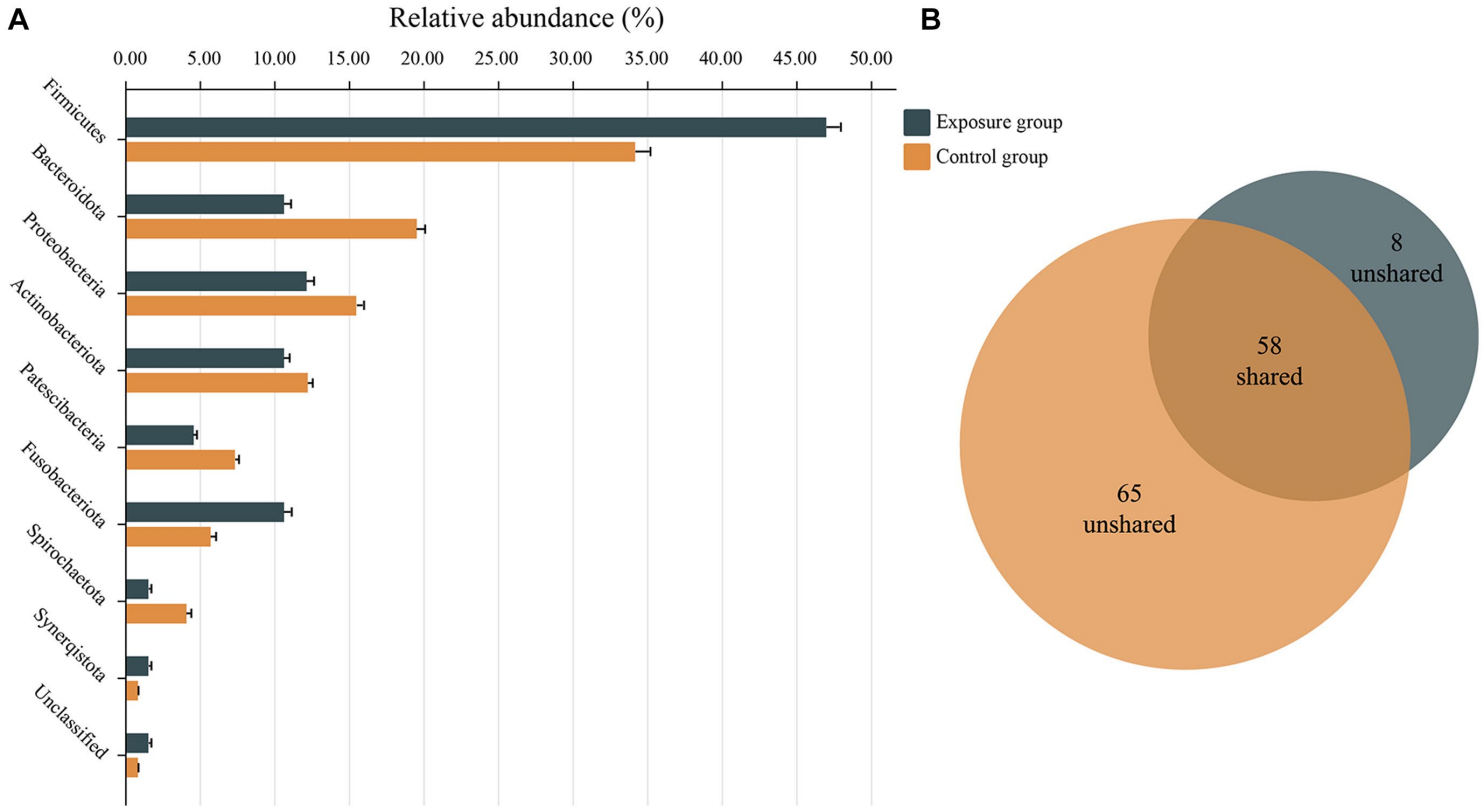
Relative abundances of different nodes in networks of the exposure and control groups. **(A)** Relative abundances of nodes belonging to different phyla categories in networks of exposure and control groups. **(B)** Venn charts showing the number of nodes shared and not shared by the networks of the exposure and control groups.

The network nodes of the exposure and control groups were mostly affiliated with 8 different bacterial phyla (Figure 7A). Among these, the relative abundances of Firmicutes, Fusobacteriota, and Synerqistota were higher in the network of the exposure group than in that of the control group. In contrast, there was a higher relative abundance of Bacteroidota, Proteobacteria, Actinobacteriota, Patescibacteria, and Spirochaetota in the network of the control group. A total of 73 nodes were unshared by the two networks (Figure 7B), indicating that most buccal mucosal bacterial species had unique niches in both networks (Qi et al., 2019). Therefore, these results indicated that the taxonomic composition of nodes was very different between the two networks.

**Figure 8:**
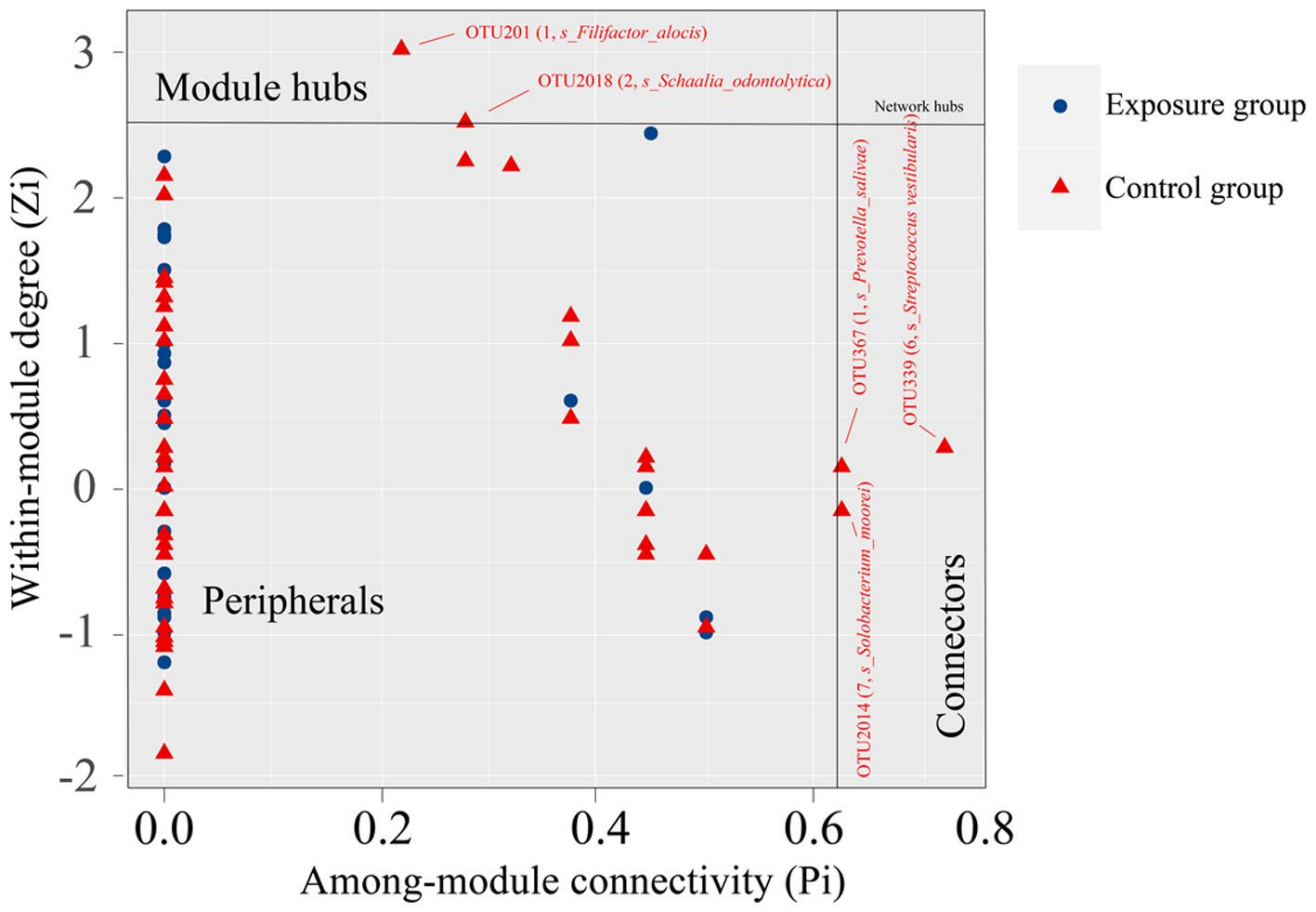
Z-P plot showing the topological roles of bacterial OTUs in the networks of the exposure and control groups. Each dot represents a bacterial OTU, and the ZP scatter plot was used to estimate the topological role of each OTU. The module hubs and connectors are labeled with OTU IDs, and in parentheses are the module IDs and species name.

We then determined the network topological roles of bacterial OTUs according to their locations in their respective modules and the extent to which they are connected to OTUs in other modules (Guimerà and Nunes Amaral, 2005). Specifically, the nodes were divided into four categories based on the indicators of within-module connectivity (Zi) and among-module connectivity (Pi): peripherals (Zi ≤ 2.5, Pi ≤0.62), connectors (Zi ≤ 2,5, Pi ≥ 0.62), module hubs (Zi ≥ 2.5, Pi ≤ 0.62), and network hubs (Zi ≥ 2.5, Pi ≥ 0.62; Guimerà and Nunes Amaral, 2005; Zhou et al., 2011; Figure 8).

Our results showed that none of the nodes within networks of the exposure and control groups were classified as network hubs, and all nodes within the network of the exposure group were peripherals with most of them (i.e., 92%) having no connections with nodes in other modules (Pi = 0). In contrast, approximately 4% of nodes within the network of the control groups were generalists (Olesen et al., 2007), with 1.6% being module hubs and 2.4% being connectors. Notably, module hubs and connectors, which are commonly thought to play an important role in the topological nature of co-occurrence networks, are considered keystone taxa (Li et al., 2017). The five keystone taxa detected in the network of the control group were affiliated with the species *Filifactor alocis* (OTU201), *Schaalia odontolytica* (OTU2018), *Prevotella salivae* (OTU367), *Solobacterium moorei* (OTU2014), and *Streptococcus vestibularis* (OTU339). Interestingly, among them, only OTU339 and OTU2018, belonging to the species *Streptococcus vestibularis* and *Schaalia odontolytica*, represented dominant taxa in the buccal mucosal bacterial community. The relative abundances of OTU339 and OTU2018 were 3.06% and 1.97%, respectively. However, the remaining three OTUs belonging to the species *Filifactor alocis*, *Prevotella salivae* and *Solobacterium moorei* only accounted for very low relative abundance in bacterial communities (averages of 0.58%, 0.13%, and 0.20%, respectively).

### 3.6. Correlations between network modules and the concentrations of heavy metals in the blood

**Figure 9:**
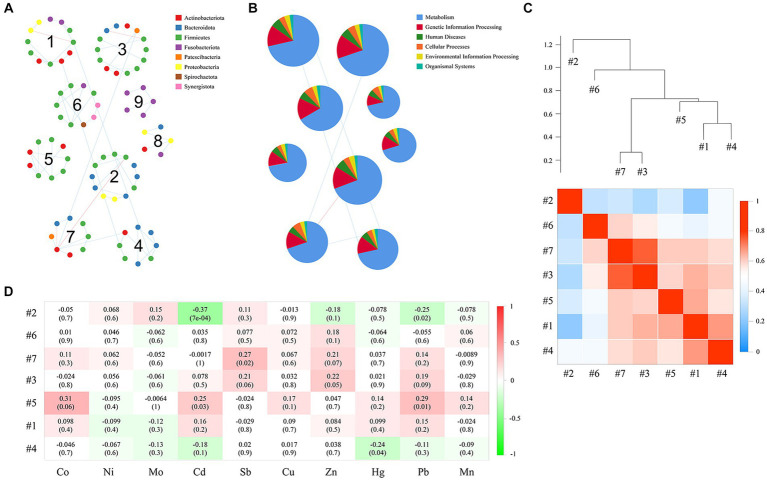
Correlation between modules, as well as the correlation of each module with the concentrations of heavy metals in the blood. Panel **(A)** presents the network module determined by the fast greedy modularity optimization method (only showing nodes larger than 4), as well as their taxonomic composition at the phylum level. Each node represents a bacterial OTU and is colored by its phylum-level taxonomic affiliation. Red lines represent negative interactions among bacterial OTUs, whereas blue lines represent positive interactions. **(B)** Relative abundance of KEGG categories of each network module. **(C)** Showing the correlations and heatmap of module eigengenes. The hierarchical clustering in the upper part is based on the Pearson correlations between module eigengenes, and the coefficient values (*r*) are shown in the lower part of the figure. **(D)** The Pearson correlations between module eigengenes and the content of heavy metals in blood. *r* in different colors, and the right side of the legend is the color range of different *r* values. The numbers represent the correlation coefficient (*r*) and significance (*P*) in parentheses.

We further tested the responses of network modules to intraexposure (i.e., the heavy metals in blood) using a subdataset that included 79 subjects. The topological properties of the empirical network are shown in Supplementary Table S4. There were obvious differences in both the average clustering coefficient (avgCC) and average path distance (GD) between the empirical and random networks (Supplementary Table S5). The empirical network had 112 nodes and 101 edges (average degree of 1.804 and average path distance of 5.537). For the network edges, the proportion of positive interactions was higher than that of negative interactions (94.06% vs. 5.94%; Supplementary Figure S4). There were 9 modules (with >4 nodes) observed in the network (Figure 9A). After modules were determined, we further used eigengene analysis to reveal the higher order organizations in the network structure (Langfelder and Horvath, 2007). Our results indicated that many sets of modules eigengenes were closely correlated with each other and were clustered together as supergroups, such as #3 and #7 and #1, #4 and #5 (Figure 9C). More importantly, we observed significant correlations between several modules and the concentrations of heavy metals in the blood (Figure 9D). Specifically, the contents of Cd and Pb were significantly and negatively correlated with module #2 (both *p* ≤ 0.02) and positively correlated with module #5 (both *p* ≤ 0.03). Module #7 was positively correlated with the concentration of Sb in the blood (*p* = 0.02), and module #4 was negatively correlated with that of Hg (*p* = 0.04). Collectively, these results suggest that different network modules respond differently to the concentrations of heavy metals in the blood, and changes in heavy metal content may have a significant impact on members of certain modules (such as #2, #4, #5, and #7).

### 3.7. Functional predictions of network modules

We used the PICRUSt pipeline to predict the bacterial gene functions for the main network modules based on KEGG metabolic pathways (Figure 9B). The predominant functions were those related to metabolism and the processing of genetic information. Our results further showed that genes related to amino acid metabolism, carbohydrate metabolism, metabolism of cofactors and vitamins, metabolism of other amino acids, and replication and repair were richest in the categories of the level 2 KEGG pathway (Supplementary Figure S5). Interestingly, some functions were unique to individual modules, such as chemical structure transformation maps, which were unique to module 3 and module 9; substance dependence, which was unique to module 5 and module 9; and the circulatory system, which was detected only in module 9. Additionally, we also calculated the correlations between the contents of heavy metals in the blood and the relative abundance of the level 2 KEGG pathway of some network modules (i.e., #2, #4, #5, and #7; Supplementary Figure S6). Our results showed that among the gene functions that were associated with the contents of heavy metals, the strongest positive correlation between Cd and Pb was cell motility, and the most negative correlations were glycan biosynthesis and metabolism and the digestive system. The strongest positive correlation with Sb was in the digestive system, and the strongest negative correlation with Hg was in cancer: specific types.

## 4. Discussion

The oral cavity, as the beginning of the digestive tract, contains a complex population of microbes (Lamont et al., 2018; Zhang Y. et al., 2018). As part of the microbial community, the microbes of the buccal mucosa play an important role in human health (Du et al., 2020; Jung and Jang, 2022), but the impact of the environment, diet, and lifestyle habits on its composition is still poorly understood. MiSeq 16S rRNA gene sequencing was performed in this study on buccal mucosal samples from a total of 137 residents living in two villages. In summary, we found differences in microbial diversity, community composition, and co-occurrence patterns between the two groups.

Microbiota analysis showed that the structure and abundance of the buccal mucosal bacteria were significantly different between the exposure and control groups. The Sobs, Ace, and Chao1 indices in the control group are significantly higher than those in the exposure group, indicating that the species richness of buccal mucosal bacteria in residents living in the contaminated area was diminished. However, the Shannon and Simpson indices show no significant differences between the two groups. We speculate that this is because, even though the number of species has decreased in the exposure group, the relative abundance distribution of species remains stable between the two groups, and there are no significant dominant species. According to the PCoA based on the weighted and unweighted UniFrac distance analysis, the microbiota distribution varied significantly between the two groups (both *p* = 0.001). These results were consistent with previous studies showing that metal exposure can alter the composition of the oral bacterial spectrum (Eshed et al., 2012; Espinosa-Cristóbal et al., 2013; Khan et al., 2013), abundance, and diversity (Youravong et al., 2011; Davis et al., 2020). In terms of the composition of the microbiota, Firmicutes, Actinobacteriota, Proteobacteria, Fusobacteriota, and Bacteroidota were the dominant phyla. This is consistent with the dominant phyla of the oral microbiome identified by the Human Microbiome Project (HMP), indicating the reliability of the results (Human Microbiome Project Consortium, 2012). Firmicutes was the most abundant phylum in the buccal mucosa, although the relative abundance in the exposed group was significantly lower than that in the control group. At the genus level, we found that *Rhodococcus*, *Delftia*, *Fusobacterium*, and *Peptostreptococcus* were significantly enriched in the exposure group, whereas the relative abundances of *Streptococcus*, *Gemella*, *Prevotella*, *Granulicatella*, and *Porphyromonas* were significantly reduced in that group. This result confirmed the idea that the variation in bacterial abundance may be associated with metal exposure and that exogenous factors control which bacteria may settle, grow, and develop in the dominant population (Sedghi et al., 2021; Akimbekov et al., 2022). Correlation analysis showed that the majority of genera that exhibited a significant increase in relative abundance in the exposed group had positive correlations with the concentration of heavy metals in the blood, such as between *Rhodococcus* and *Delftia* with Cd and Pb. Conversely, in the control group, most genera with a significant increase in relative abundance were negatively correlated with the concentration of heavy metals in blood such as Cd with *Streptococcus, Porphyromonas*, and *Granulicatella*. Therefore, we speculated that long-term residence in areas contaminated with heavy metals leads to heavy metal accumulation in the body and further affects the microbial community structure of the buccal mucosa.

Oral lichen planus (OLP) is considered to be a chronic inflammatory disease associated with the buccal mucosa (Jung and Jang, 2022), and although the mechanism and cause of OLP are not yet explained, studies have found that the bacteria that settle on the surface of the buccal mucosa are related to OLP (Choi et al., 2016; He et al., 2017). In their study of the composition of the bacteria in the buccal mucosa of OLP patients and healthy controls, Hijazi et al. found that although there were no significant differences in the bacterial diversity between the two groups, alpha diversity decreased as the severity of OLP increased (Hijazi et al., 2020). Additionally, studies have shown that people with OLP have a more diverse microbiota in their buccal mucosa than healthy controls (He et al., 2017; Baek and Choi, 2018). In summary, although some studies report that the bacterial composition of healthy controls was similar to that of OLP patients (Hijazi et al., 2020), most of the studies on the microbial composition of the buccal mucosa have found that the bacterial structure of OLP patients can be distinguished from that of healthy controls (Du et al., 2020; Wang et al., 2020). In a previous study, a total of 19 different genera were found to have significant differences in abundance between OLP patients and healthy controls, with a significant increase in *Fusobacterium*, *Leptotrichia*, and *Lautropia* in patients, while *Streptococcus* was lower compared to the healthy control group (He et al., 2017). We created a combinatorial marker panel composed of four genera (*Fusobacterium*, *Leptotrichia*, *Lautropia*, and *Streptococcus*) and employed random forest analysis to investigate whether these genera could differentiate between the exposed and control groups (Supplementary Figure S7A). In the receiver-operating characteristic (ROC) curve, this result highlights the diagnostic potential of the combinatorial marker panel (AUC = 0.81, 95%CI: 0.73–0.89). It is reported that *Streptococcus* was closely associated with the development of oral cancer, and the relative abundances of *Streptococcus anginosus* and *S. gordonii* were significantly enriched in the buccal mucosa in patients with oral squamous cell carcinoma (OSCC; Karpiński, 2019). Moreover, *Streptococcus* (including *S. salivarius*, *S. mutans*, *S. milleri/anginosus*, and *S. mitis*) is alpha-hemolytic and opportunistic pathogenic (Nobbs et al., 2009). In this study, we found that the abundance of *S. anginosus* increased significantly in the exposure group, and that of *S. gordonii* and *S. mutans* increased but not significantly. However, in the ROC analysis, the combinatorial marker panel composed of *S. anginosus*, *S. gordonii*, and *S. mutans* cannot accurately distinguish between the exposure group and control group (AUC = 0.58, 95%CI: 0.48–0.868; Supplementary Figure S7B). Thus, our findings reveal that people who live in different environments have different microbial communities in the buccal mucosa and that living in areas contaminated with heavy metals may increase the risk of several diseases, including oral lichen planus.

PICRUSt analysis was used to predict the function of bacterial communities. In this study, there were significant differences in the metabolic function of buccal mucosal bacteria in the two groups, with most functions being upregulated in the exposure group. Metal ions may improve the adaptability of bacteria to different environments by regulating the function of bacterial cells in terms of substance dependence, xenobiotic biodegradation and metabolism, lipid metabolism, the excretory system, and the sensory system. According to findings from a previous study, during succession under the impact of the environment, microbes can develop adaptive mechanisms (Zhu et al., 2019). Due to the limitations of PICRUSt functional predictions (Langille et al., 2013), this study is only a preliminary prediction of bacterial function, and further verification should be carried out in future studies using methods such as metagenomics to better understand the function of buccal mucosal bacteria from different populations.

Based on high-throughput 16S rRNA sequencing data, we constructed microbial networks in this study. Most previous studies only analyzed the community composition, abundance, and diversity of oral microbes (Dong et al., 2021; Zhang et al., 2023), but the interactions between microbial species are critical to ecosystem stability. Therefore, microbial networks, as a new approach to analyzing the interactions between microbiota populations, can help us better understand the changes between the exposure and control groups.

Through bacterial molecular ecological network analysis, we observed that the network of the control group exhibited a greater number of interacting microbial species compared to the exposed group. In general, more interactive bacteria are present in the network, suggesting that there is more metabolism and information exchange between species, which allows the network work more efficiently (Faust and Raes, 2012). Modularity is one of the key topological features of network structures (Newman, 2006) and nodes in the same module usually have similar functions, metabolic pathways, niches, or phenotypic features (Qin et al., 2012; Layeghifard et al., 2017; Lurgi et al., 2019). In this study, both the number of modules and nodes within each module were found to be lower in the network of the exposure group than in the network of the control group, which means that the microbial network of the control group has a higher complexity and ecological diversity, and the interactions between microbes are more complex and tighter. Furthermore, a higher average connectivity implies a network of greater complexity (Qi et al., 2019). The average degree values for the exposure and control groups were 1.697 and 2.195, respectively. All of the aforementioned results indicated that exposure to heavy metals would reduce the complexity of the buccal mucosal bacterial network.

Keystone taxa were believed to play an important role in the network, making it more stable and ordered (Qi et al., 2019). Studies have shown that keystone taxa are important for the stability of ecosystems, and their extinction may lead to the fragmentation of the entire microbiome (Lupatini et al., 2014). In the network of the control group, we found 5 keystone taxa, while in the exposure group all nodes were identified as peripherals. In addition, the role of microbial species changed in the network of the exposure group compared to the control group. For example, module hubs in the network of the control group (OTU201 and OTU2018) and connectors (OTU367, OTU2014, and OTU339) were converted to peripherals in the exposure group. Shifts in the roles of these keystone taxa may lead to instability of the exposure group network and a weakening of the ability to suppress the growth of pathogens (such as *S. anginosus*, *S. gordonii*, and *P. gingivalis*). Interestingly, of the keystone taxa found in the control group, *Filifactoralocis*, *Prevotella salivae*, and *Solobacterium moorei* together accounted for less than 1% of the total relative abundance. These findings suggest that these relatively low-abundance bacteria occupy a very important place in the ecological network and therefore may exert a greater influence on microbial structure and function than some bacteria with relatively high abundances, despite the fact that their presence can have either beneficial or detrimental effects on humans and human activities (Zhang S. et al., 2018; Qi et al., 2019). Furthermore, we did not identify any keystone taxa in the network of the exposure group, indicating that heavy metal exposure would alter the initial structure of the network, causing the loss of the activities of taxa that were in key positions before and thereby making the entire network more vulnerable to damage. We also found that the concentrations of Cd, Pb, Sb, and Hg in blood were correlated with the microbial network structure of the buccal mucosa. Previous studies have shown that heavy metal ions, especially divalent ions such as lead and cadmium, affect the growth and vitality of oral bacterial communities (Youravong et al., 2011; Breton et al., 2013; Steiger et al., 2020). Thus, we speculate that Cd, Pb, Sb, and Hg in the blood are the main drivers of the bacterial network and may adversely affect the stability of the microbial network of the buccal mucosa in residents with long-term exposure to heavy metals.

## 5. Conclusion

1. Long-term exposure to multiple metals perturbs normal buccal mucosal bacterial communities in inhabitants of contaminated areas and may increase their risk of developing a variety of diseases, including OLP.
2. The concentrations of heavy metals (Pb, Cd, Hg, and Mo) in the blood are associated with the growth of *Rhodococcus*, *Delftia*, *Porphyromona*s, and *Gemella*.
3. Long-term exposure to metals, reduces the complexity and stability of the microbial network of the buccal mucosa.
4. As the main drivers of the network, Pb, Cd, Hg, and Mo in the blood can adversely affect the microbial network of the buccal mucosa in residents with long-term exposure to heavy metals.
5. Some low-abundance bacteria may exert a greater influence on microbial structure than some bacteria with a relatively high abundance.

## Data availability statement

The datasets presented in this study can be found in online repositories. The names of the repository/repositories and accession number(s) can be found at: https://www.ncbi.nlm.nih.gov/, PRJNA979792.

## Ethics statement

The studies involving humans were approved by Ethical Committees of the Public Health School of Lanzhou University. The studies were conducted in accordance with the local legislation and institutional requirements. The participants provided their written informed consent to participate in this study.

## Author contributions

SP: Data curation, Software, Writing – original draft. LF: Writing – review & editing. YZ: Data curation, Investigation, Methodology, Writing – review & editing. JLiu: Investigation, Methodology, Software, Writing – review & editing. JLi: Data curation, Software, Writing – review & editing. QZ: Data curation, Software, Writing – review & editing. XL: Conceptualization, Writing – review & editing. BL: Conceptualization, Writing – review & editing. HL: Conceptualization, Writing – review & editing. WH: Data curation, Methodology, Software, Writing – review & editing. JN: Conceptualization, Investigation, Writing – review & editing. TT: Conceptualization, Investigation, Writing – original draft. YR: Conceptualization, Investigation, Writing – review & editing.

## Footnotes

^1^ http://mem.rcees.ac.cn:8081

^2^ https://gephi.org/

^3^ https://cytoscape.org/

^4^ https://github.com/picrust/picrust2
