# Supplementary material for: Effects of long-term metal exposure on the structure and co-occurrence patterns of the oral microbiota of residents around a mining area
Source: Front Microbiol. 2023 Oct 19;14:1264619. doi: 10.3389/fmicb.2023.1264619 (PMC10620801; doi:10.3389/fmicb.2023.1264619)
Supplement: Supplementary file 1 [file Data_Sheet_1.docx]

Supplementary Material

**Figures**

**
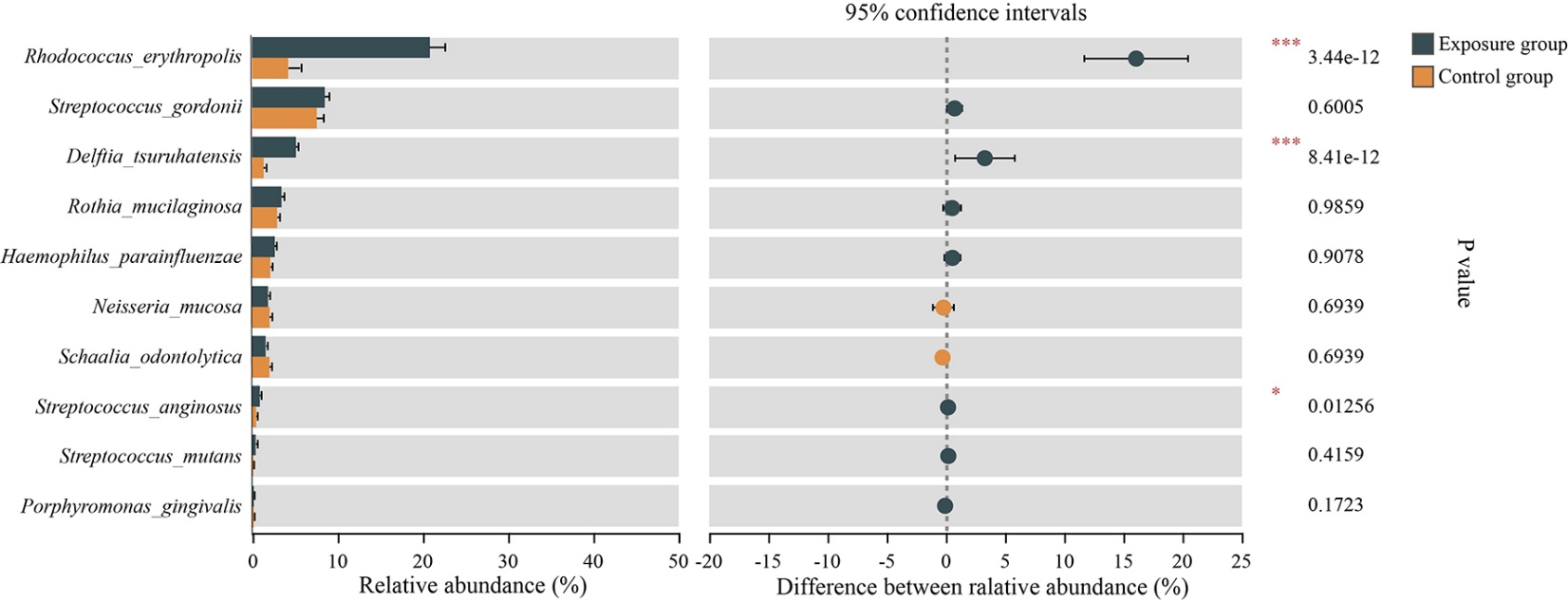
**

**Supplementary Figure 1.** Compositional differences in bacterial communities of the buccal mucosa at species levels. *P* value was calculated using the Wilcoxon rank-sum test and adjusted by using false discovery rate. **P* < 0.05; ***P* < 0.01; ****P* < 0.001.


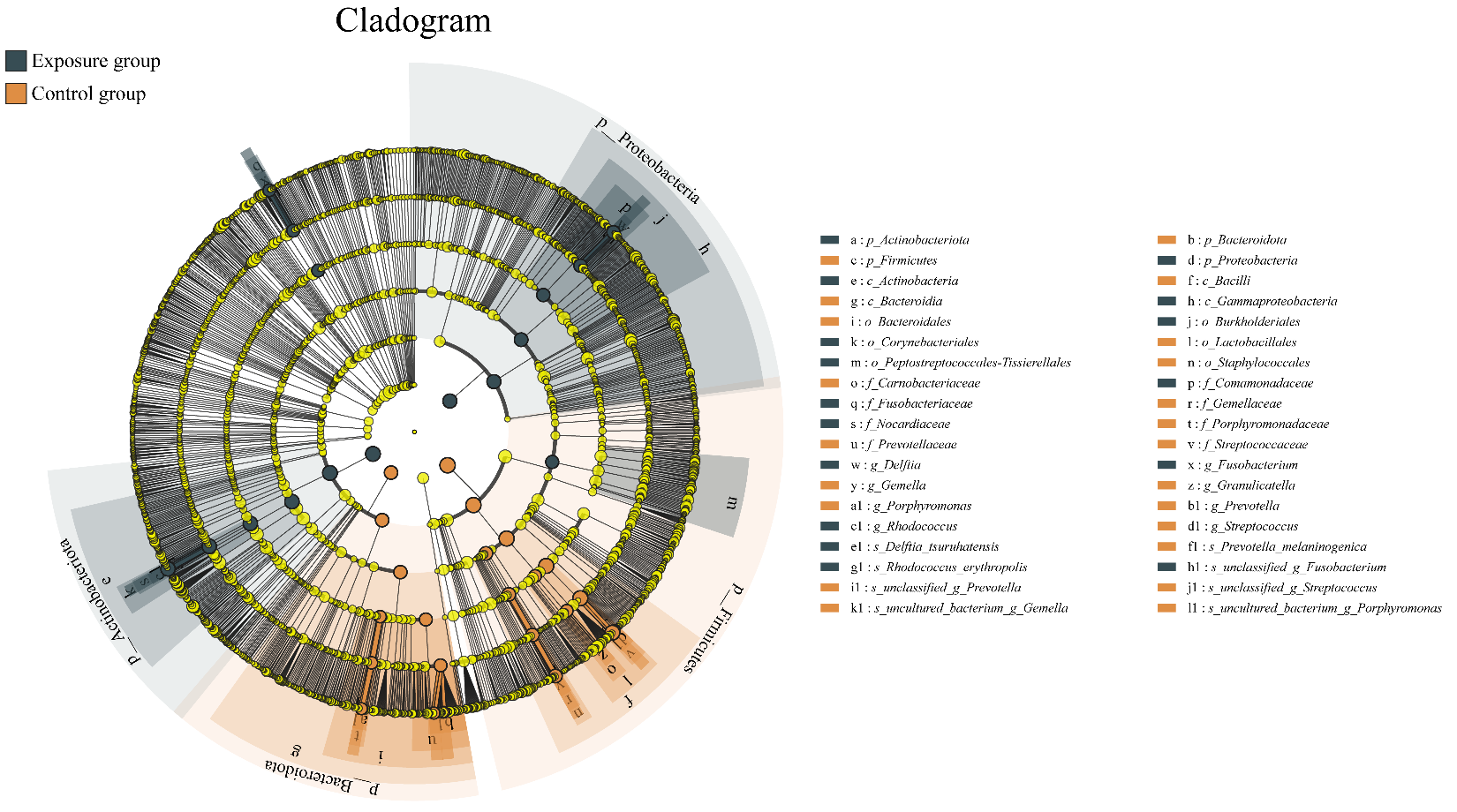


**Supplementary Figure 2.** The LEfSe analysis shows the phylogenetic distribution of the bacterial lineages among two groups. The circles that radiate outward from the center of the branch diagram stand in for the various levels of classification, from phylum to species; each small circle at a given level of classification represents a classification at that level, and the diameter of the small circle is proportional to the relative abundance.


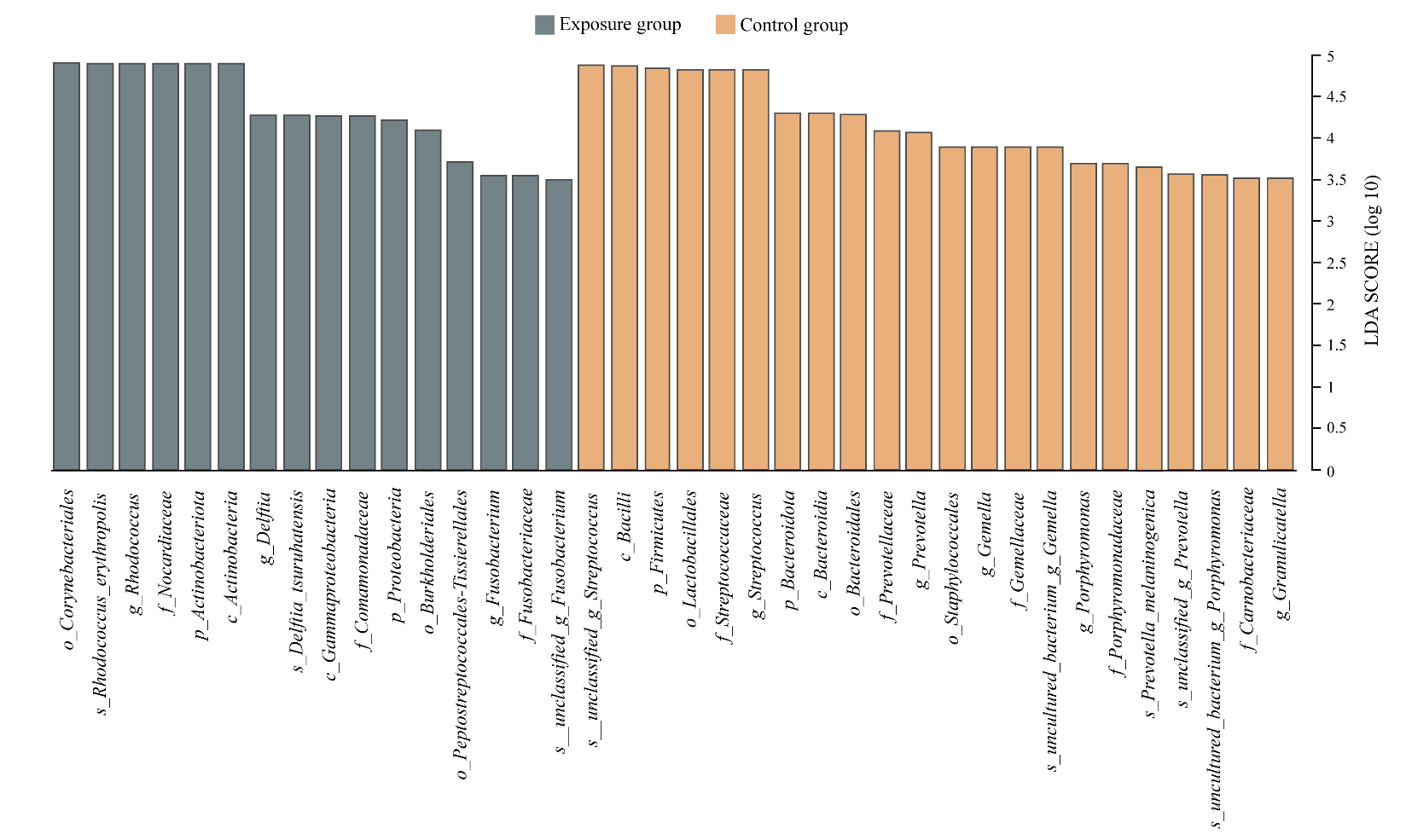


**Supplementary Figure 3.** The significant taxa were examined using an LEfSe analysis and shown using a histogram. The histogram visually displays the LDA scores for the differentially abundant features between the two groups.


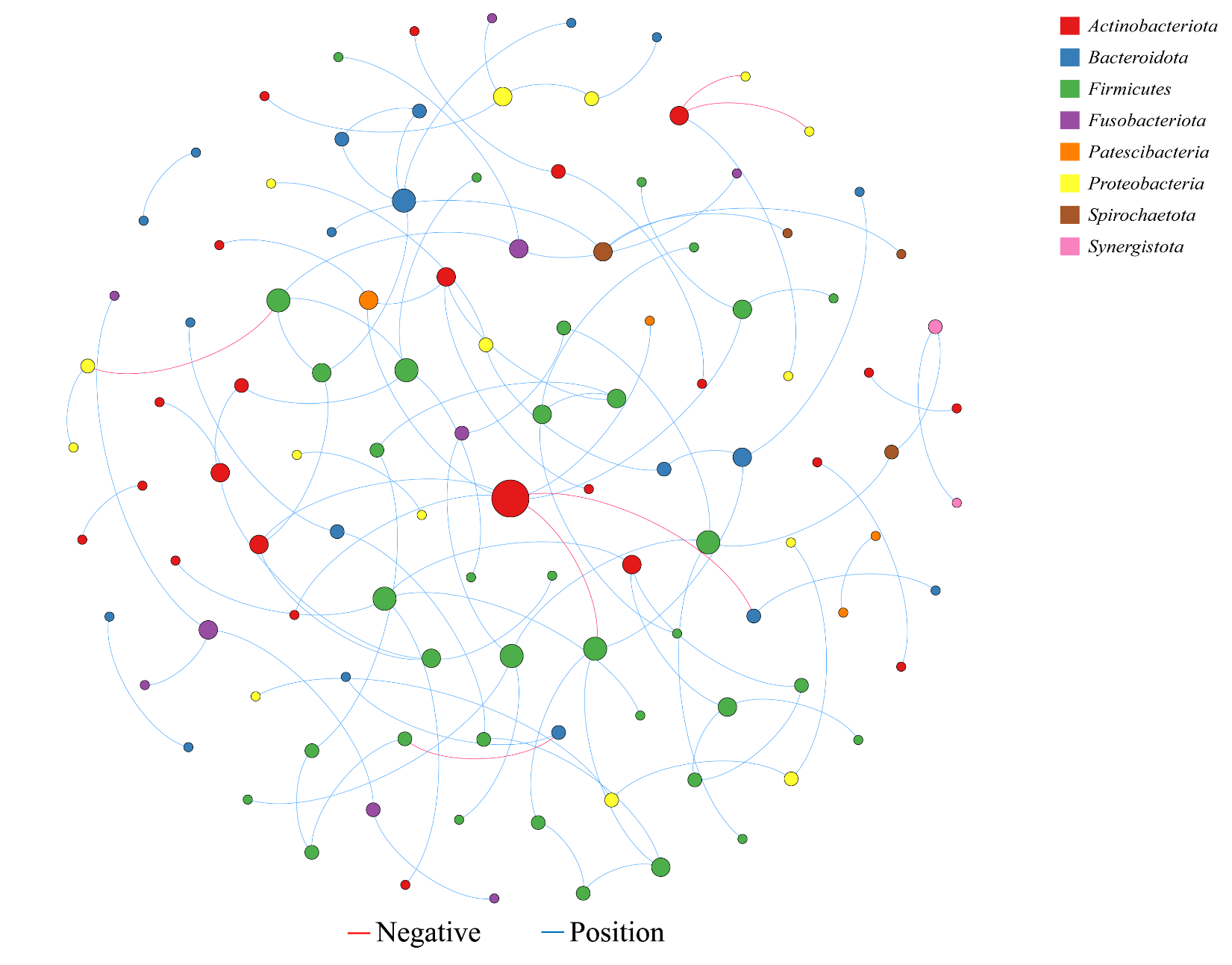


**Supplementary Figure 4.** Molecular ecological networks were built on the basis of correlation among bacterial OTUs. Node sizes are proportional to the number of connections. Each node represents a bacterial OTU and is coloured by its phylum-level taxonomic affiliation. Red lines represent negative interactions among bacterial OTUs, whereas blue lines represent positive interactions.


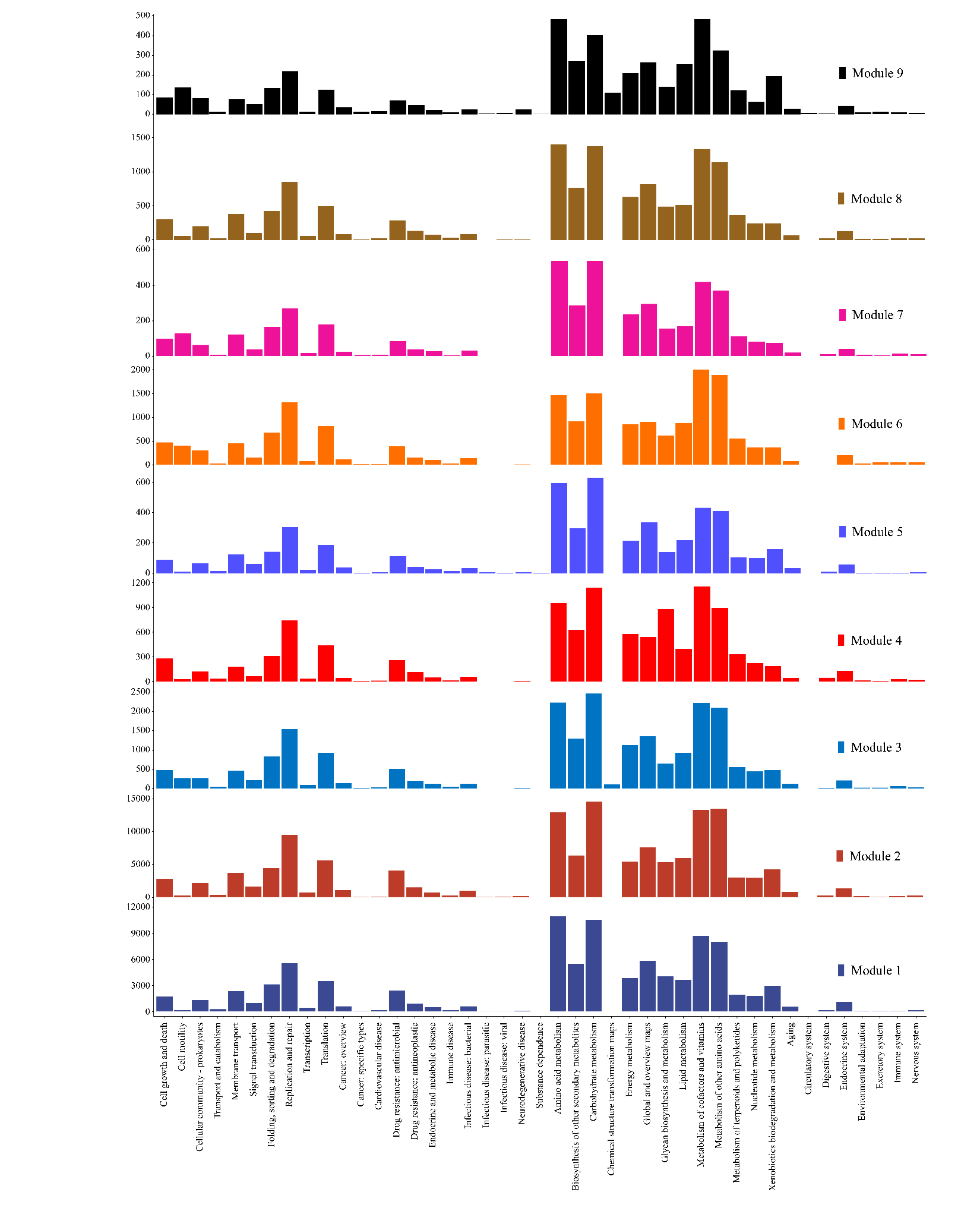


**Supplementary Figure 5.** The abundance of the level 2 KEGG pathway of the network modules.


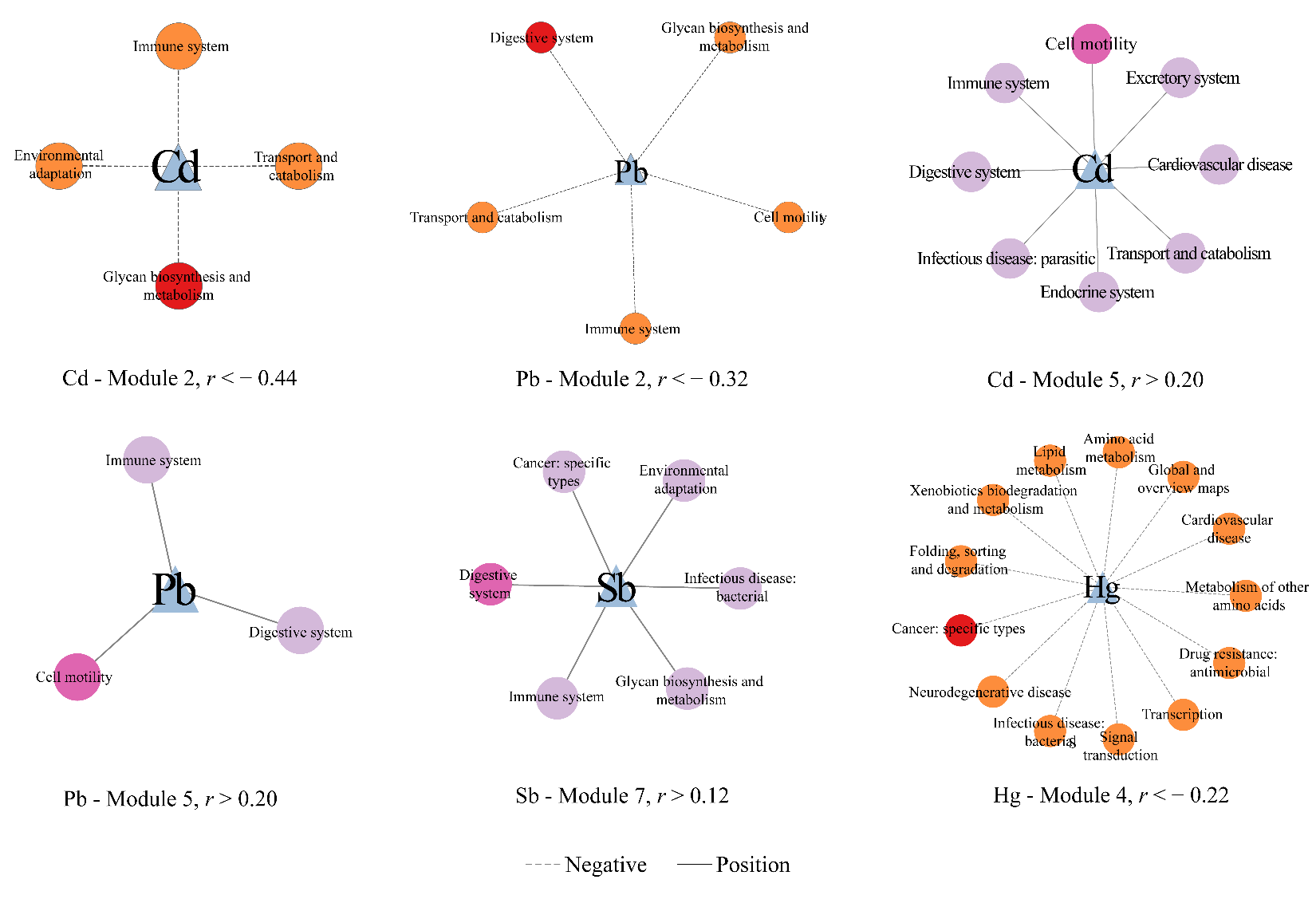


**Supplementary Figure 6.** Spearmen correlation analysis was used to assess connections between the blood variables and the abundance of the level 2 KEGG pathway of network modules (*P* < 0.05). The pathways within the module with the strongest correlation to blood variables are marked in dark red or purple.

**
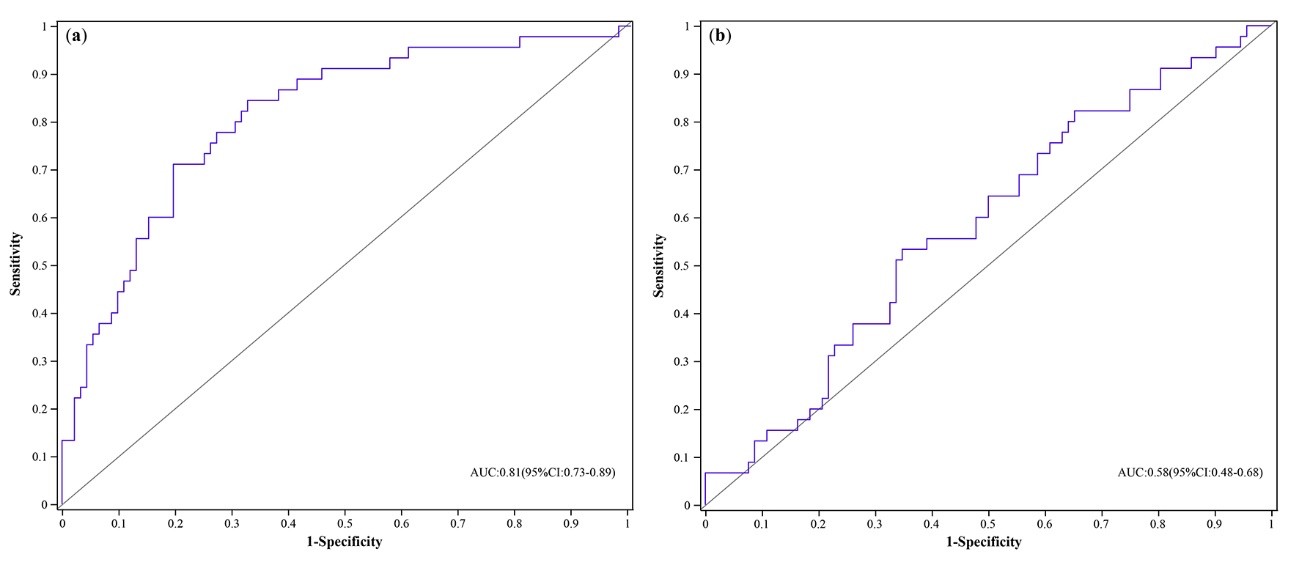
**

**Supplementary Figure 7. Area under the curve of the combinatorial marker between the exposure and control groups.** AUC, area under the curve; ROC, receiver‐operating characteristic.

**Table**

**Supplementary Table 1.** The concentration of metals in soils between contaminated and control areas (unit: mg/kg).

| Element | B (n = 6) | Y (n = 7) | *P*-value |
| --- | --- | --- | --- |
| Mo | 0.27 ± 0.06 | 0.09 ± 0.01 | 0.001* |
| Cd | 7.81 ± 5.21 | 0.11 ± 0.01 | 0.001* |
| Sb | 0.02 ± 0.00 | 0.00 ± 0.00 | 0.001* |
| Cu | 96.99 ± 25.45 | 18.00 ± 1.00 | 0.001* |
| Zn | 527.36 ± 313.25 | 47.63 ± 3.68 | 0.001* |
| Pb | 168.36 ± 49.94 | 12.98 ± 0.82 | 0.001* |
| Hg | 0.52 ± 0.15 | 0.02 ± 0.01 | 0.002* |
| Co | 8.29 ± 0.96 | 9.66 ± 0.64 | 0.181 |
| Ni | 31.31 ± 4.70 | 24.25 ± 1.54 | 0.234 |
| Mn | 514.00 ± 51.79 | 431.61 ± 33.06 | 0.534 |

B, contaminated areas; Y, control areas. Data were shown as mean ± SE. **P* < 0.01.

**Supplementary Table 2.** The concentration of metals in the blood of subjects living in both contaminated and control areas (unit: ng/ml).

| Element | B (n = 63) | Y (n = 16) | *P*-value |
| --- | --- | --- | --- |
| Mo | 2.34 ± 0.25 | 2.15 ± 0.53 | 0.506 |
| Cd | 8.62 ± 0.65 | 1.91 ± 0.43 | 0.000*** |
| Sb | 0.10 ± 0.01 | 0.10 ± 0.02 | 0.966 |
| Cu | 816.78 ± 14.06 | 776.46 ± 34.43 | 0.145 |
| Zn | 5831.07 ± 117.48 | 5238.74 ± 196.88 | 0.025* |
| Pb | 52.55 ± 2.67 | 18.42 ± 1.13 | 0.000*** |
| Hg | 0.56 ± 0.03 | 0.42 ± 0.04 | 0.015* |
| Co | 0.50 ± 0.07 | 0.37 ± 0.04 | 0.908 |
| Ni | 4.18 ± 0.77 | 4.89 ± 2.27 | 0.351 |
| Mn | 18.83 ± 1.33 | 15.62 ± 0.93 | 0.390 |

B, contaminated areas; Y, control areas. Data were shown as mean ± SE. **P* < 0.05, ***P* < 0.01, ****P* < 0.001.

**Supplementary Table 3.** Comparison in topological properties of empirical and randomized networks of microbial communities based on student’s t-test.

| Index | avgCC | | GD | | Modularity | |
| --- | --- | --- | --- | --- | --- | --- |
| Group | Exposure | Control | Exposure | Control | Exposure | Control |
| Empirical network | 0.075 | 0.072 | 3.021 | 6.838 | 0.848 | 0.793 |
| Random network | 0 ± 0.005 | 0 ± 0.002 | 4.611 ± 0.928 | 5.814 ± 0.331 | 0.799 ± 0.018 | 0.725 ± 0.014 |
| Student’s *t* value | 150.0000 | 360.000 | 17.1336 | 30.9366 | 27.2222 | 45.5714 |
| *P* value | < 0.001 | < 0.001 | < 0.001 | < 0.001 | < 0.001 | < 0.001 |

**Supplementary Table 4.** Topological properties of the empirical and 100 random MENs of microbial communities. n.a denotes no data available in the random algorithm.

| Network Indexes | Empirical | Random (Mean ± SD) |
| --- | --- | --- |
| Total nodes | 112 | n.a |
| Total links | 101 | n.a |
| RMT cutoff | 0.44 | n.a |
| R square of power-law | 0.981 | n.a |
| Average degree (avgK) | 1.804 | n.a |
| Average clustering coefficient (avgCC) | 0.019 | 0 ± 0.002 |
| Average path distance (GD) | 5.537 | 6.941 ± 1.336 |
| Geodesic efficiency (E) | 0.266 | 0.221 ± 0.037 |
| Harmonic geodesic distance (HD) | 3.754 | 4.637 ± 0.703 |
| Centralization of degree (CD) | 0.047 | 0.047 ± 0 |
| Centralization of betweenness (CB) | 0.159 | 0.193 ± 0.069 |
| Centralization of stress centrality (CS) | 0.166 | 0.002 ± 0.001 |
| Centralization of eigenvector centrality (CE) | 0.944 | 0.945 ± 0.01 |
| Centralization of closeness centrality (CCL) | 0.008 | 0.01 ± 0.002 |
| Density (D) | 0.016 | 0.016 ± 0 |
| Reciprocity | 1 | 1 ± 0 |
| Transitivity (Trans) | 0.021 | 0.009 ± 0.013 |
| Connectedness (Con) | 0.251 | 0.396 ± 0.106 |
| Efficiency | 0.965 | 0.999 ± 0.001 |
| Hierarchy | 0 | 0.016 ± 0 |
| Lubness | 1 | 0.078 ± 0.026 |
| Modularity | 0.863 (20) | 0.828 ± 0.015 |

**Supplementary Table 5.** Comparison in topological properties of empirical and randomized networks of microbial communities based on student’s t-test.

| Indes | Empirical networks | Random networks | Student’s t value | P value |
| --- | --- | --- | --- | --- |
| avgCC | 0.019 | 0 ± 0.002 | 95.000 | < 0.001 |
| GD | 5.537 | 6.941 ± 1.336 | 10.5090 | < 0.001 |
